# Thermoplasmonic In Situ Fabrication of Nanohybrid Electrocatalysts over Gas Diffusion Electrodes for Enhanced H_2_O_2_ Electrosynthesis

**DOI:** 10.1021/acscatal.3c01837

**Published:** 2023-07-20

**Authors:** Yu Zhang, Luca Mascaretti, Michele Melchionna, Olivier Henrotte, Štepan Kment, Paolo Fornasiero, Alberto Naldoni

**Affiliations:** † Czech Advanced Technology and Research Institute, Regional Centre of Advanced Technologies and Materials, Palacký University Olomouc, Šlechtitelů 27, 78371 Olomouc, Czech Republic; ‡ Department of Chemical and Pharmaceutical Sciences, ICCOM-CNR Trieste Research Unit, INSTM-Trieste, Center for Energy, Environment and Transport Giacomo Ciamician, University of Trieste, Via L. Giorgieri 1, 34127 Trieste, Italy; § Nanotechnology Centre, Centre of Energy and Environmental Technologies, VŠB—Technical University of Ostrava, 17. listopadu 2172/15, Poruba, 708 00 Ostrava, Czech Republic; ∥ Department of Chemistry and NIS Centre, University of Turin, 10125 Turin, Italy

**Keywords:** thermoplasmonics, oxygen reduction reaction, plasmonics, titanium nitride, nanohybrids, electrocatalysis

## Abstract

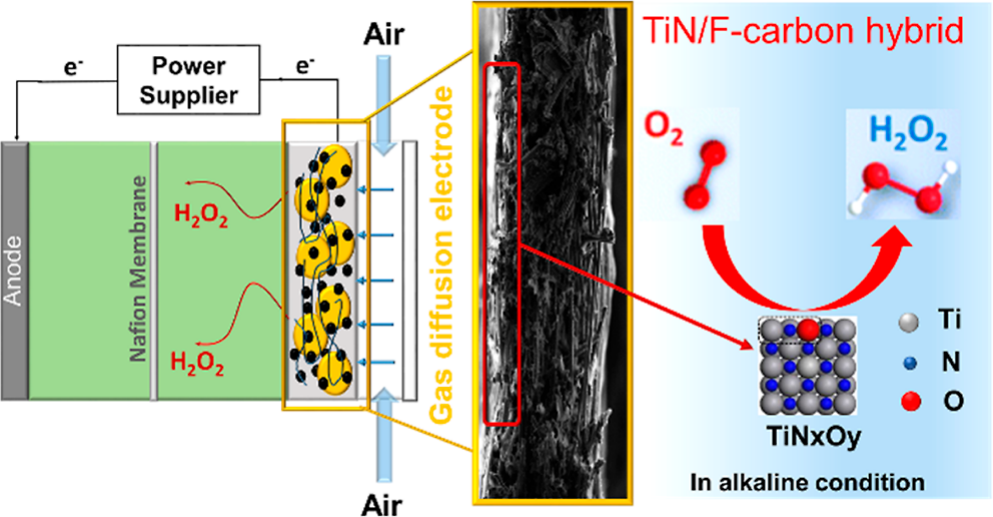

Large-scale development of electrochemical cells is currently hindered by the lack of Earth-abundant electrocatalysts with high catalytic activity, product selectivity, and interfacial mass transfer. Herein, we developed an electrocatalyst fabrication approach which responds to these requirements by irradiating plasmonic titanium nitride (TiN) nanocubes self-assembled on a carbon gas diffusion layer in the presence of polymeric binders. The localized heating produced upon illumination creates unique conditions for the formation of TiN/F-doped carbon hybrids that show up to nearly 20 times the activity of the pristine electrodes. In alkaline conditions, they exhibit enhanced stability, a maximum H_2_O_2_ selectivity of 90%, and achieve a H_2_O_2_ productivity of 207 mmol g_TiN_^–1^ h^–1^ at 0.2 V vs RHE. A detailed electrochemical investigation with different electrode arrangements demonstrated the key role of nanocomposite formation to achieve high currents. In particular, an increased TiO_*x*_N_*y*_ surface content promoted a higher H_2_O_2_ selectivity, and fluorinated nanocarbons imparted good stability to the electrodes due to their superhydrophobic properties.

## Introduction

Increasing concerns about global warming have prompted worldwide efforts in developing innovative energy storage technologies. Electrochemistry plays a crucial role in such a transition to sustainable processes as mean of either storing electrical energy in chemical bonds generating fuels, such as in water splitting,^1,2^ CO_2_ reduction,^3,4^ hydrogen peroxide (H_2_O_2_) production,^5,6^ or generating electrical energy by consuming such fuels, such as in fuel cells^7,8^ or in metal-air batteries.^9^ Among these processes, the oxygen reduction reaction (ORR) is central for many technologies. It may occur according to a four-electron pathway (O_2_ + 4H^+^ + 4e^–^ → 2H_2_O) at the cathodic side in both fuel cells and metal-air batteries, respectively.^10^ On the other hand, the ORR can also proceed along a two-electron pathway, leading to hydrogen peroxide (O_2_ + 2H^+^ + 2e^–^ → H_2_O_2_).^11^ Although H_2_O_2_ is an undesired side product in fuel cells,^12^ its synthesis through electrochemical approach has recently attracted considerable attention due to the possibility for decentralized H_2_O_2_ production.^13−18^

While state-of-the-art cathodes for the ORR are based on Pt nanoparticles (NPs) loaded on carbon supports (Pt/C),^19^ alternative Pt-free electrodes based on doped carbon nanostructures^20−23^ or on transition-metal nitrides (TMNs) have been considered^24−27^ due to their high ORR activity maintaining low cost. Heteroatom-doped carbon catalysts, particularly in the nanostructured form, have been the subject of wide interest in H_2_O_2_ electrochemical synthesis. In this case, the presence of atoms with different electronegativity inserted within the graphitic carbon lattice may tune the charge distribution on the carbon atoms adjacent to the heteroatom. The resulting polarization favors optimum O_2_ adsorption, triggering the selective reduction pathway. While nitrogen is the most investigated heteroatom, various non-metal elements have been screened and their effect evaluated in the realm of 2e^–^ ORR, including fluorine.^28,29^ Notably, F-doped carbons show a high positive charge over the carbon framework due to the highest electronegativity of fluorine atoms, which show great potential for ORR,^30^ especially facilitating the desorption of the *OOH intermediate.^31^ Besides doped carbon, TMNs are well-suited as electrocatalysts due to their exceptional electrical conductivity, chemical stability, wear resistance, and mechanical robustness.^32−34^ The limited performance of TMNs compared to commercial Pt/C can be improved through doping^35,36^ or by partial oxidation, leading to oxynitride phases.^27,37−42^ Furthermore, a fully or partially oxidized surface layer can increase the selectivity toward the 2e^–^ ORR over the 4e^–^ ORR, as shown in the case of titanium nitride/oxynitride (TiN/TiO_*x*_N_*y*_) electrocatalysts.^38,40,43,44^

Apart from the field of electrochemistry, the refractory nature of TMNs coupled with their similar optical properties to Au has led to their implementation as alternative plasmonic materials to substitute coinage metals, especially in the so-called field of thermoplasmonics, which makes use of the photothermal heat generated after surface plasmon dissipation and hot carrier thermalization.^32,33,45−47^ The photothermal properties of TMNs have been shown to be beneficial in promoting chemical transformations, in material synthesis,^48−51^ solar desalination,^47,52^ catalysis,^53−55^ and as nanosources of heat.^47^ In particular, electromagnetic hotspots in plasmonic materials enable the generation of significant local heating with characteristic ultrafast heating/cooling rates of ∼25 °C s^–1^ for TiN nanostructures, thus producing unique conditions for nanomaterial synthesis.^45^

Gas diffusion electrodes (GDEs) are chosen as they represent a benchmark to industrially scaling electrochemical processes by allowing substantially higher reaction currents compared to traditional electrode configurations.^56,57^ However, GDEs tend to lose their hydrophobicity during longtime electrolysis, leading to the flooding of the electrodes and, consequently, a decrease in O_2_ mass transport and performance over time. In order to overcome these limitations, GDE functionalization to generate engineered reaction microenvironment^58^ and superhydrophobic surfaces,^59,60^ which ensure stable solid–gas–liquid triple phase interfaces, has been proposed as effective strategies. For example, surface fluorination is an efficient approach to tuning surface wettability.^61−65^ In particular, Nafion polymer,^66^ polytetrafluoroethylene (PTFE),^64^ and others fluorinated organic binders^30^ have been proposed as precursors to synthesize F-doped nanocarbons. However, the high cost of Nafion raises questions about the scalability of the approach, while PTFE can be a more viable solution as demonstrated for electrocatalytic CO_2_ reduction.^67^ Thus, an efficient and more convenient strategy for the synthesis of F-doped nanocarbons to fabricate stable GDEs is still a challenge.

Here, we present a thermoplasmonic synthesis approach, leading to TiN/F-doped carbon hybrid cathodes for the ORR with enhanced activity and selectivity toward H_2_O_2_ electrosynthesis. In such nanohybrids, each material provided functional properties to boost the performance of the cathodes that were directly assembled as GDEs using natural air to feed the ORR. In particular, plasmonic TiN in the form of nanocubes was chosen as an electrochemically active phase and to provide plasmonic local heating upon irradiation. Carbon fiber was chosen as a catalyst backing layer to form GDE to enhance O_2_ mass transport and improve the electrochemical activity. Surface fluorination was then achieved through the decomposition of F-containing organic binders upon irradiation to both induce enhanced activity and higher hydrophobicity to the hybrid electrodes, where the latter is paramount to avoid flooding of GDEs and ensure a constant supply of O_2_.^59,60,64^ PTFE was chosen as a low cost fluorinated alternative to Nafion, whose final nanohybrid electrocatalyst performance were compared with a non-fluorinated binder. The unique conditions created during thermoplasmonic synthesis led to the TiN surface enrichment in Ti^(III)^O_*x*_N_*y*_ species, which favored the 2e^–^ ORR over the 4e^–^ ORR, and the formation of F-doped nanocarbons, which ensured a stable three-phase interface during the electrochemical reactions. The so-realized hybrid TiN/F-carbon GDE exhibited an ORR current of ∼25 mA cm^–2^ at 0.1 V vs RHE and a maximum faradaic efficiency (FE) toward H_2_O_2_ production of ∼75% at 0.5 V vs RHE. This work provides a new paradigm to enhance the electrochemical activity of nanohybrids toward H_2_O_2_ and offers a scalable and universal plasmon-assisted fabrication route toward in situ functionalization of GDEs, which could be easily implemented into electrochemical cells for the synthesis of a wide variety of commodity chemicals.

## Experimental Section

### Materials and Chemicals

Toray carbon paper (TGP-H-60) without PTFE treatment was purchased from Alfa Aesar. Sulfuric acid (95–98% H_2_SO_4_), polytetrafluoroethylene (PTFE) 60 wt % dispersion in water, polyvinyl alcohol (PVA) powder *M*_w_ = 146,000–186,000, KOH pellet, and Nafion perfluorinated resin solution (5 wt %) were purchased from Sigma-Aldrich. 30 wt % H_2_O_2_ was purchased from Lanchner. Ethanol (absolute) was purchased from Penta. Commercial TiN nanocubes were obtained from PlasmaChem GmbH (average size ∼ 50 nm). All aqueous solutions were prepared with ultrapure water (18 MΩ cm^–1^). All the chemicals and materials were used without further treatment.

### Electrode Preparation

The TiN/binder ink was prepared by adding 10 mg of TiN and 20 μL of 5 wt % binder solution Nafion, PTFE, and PVA solution to ethanol to reach a concentration of 10 mg mL^–1^, then sonicating for 30 min. 50 μL of the precursor ink was drop-cast on each side over a 5 × 27 mm Toray carbon paper and heated at 70 °C by using a heating plate to enhance the solvent evaporation. The same procedure was applied for the fabrication of self-supported GDEs, enlarging the coated area to 7 × 27 mm and drop-casting on each side of the Toray paper 70 μL of the precursor ink. The electrodes were then put into a 50 mL Schlenk tube glass, which was evacuated with a vacuum pump (EDWARDS model E2M1.5) for 30 min. Then, the sample was irradiated from one side by means of solar-simulated light provided by a 1000 W solar simulator (Sciencetech A4 Lightline C250) equipped with a xenon short arc lamp (Osram XBO 1000W/HS OFR). Two lenses (i.e., a fused silica plano-convex lens, Thorlabs LA4984, and a Fresnel lens, Thorlabs FRP251) were employed to focus the light beam down to a circular spot of 1 cm diameter with an average intensity of 1.5 W cm^–2^ (i.e., 15 Suns), which was measured with a thermopile detector (Standa 11UP19K-30 H-H5) prior to every experiments. The scheme of the set-up employed for the electrode synthesis is illustrated in Figure S1.

### Characterization

Fourier-transform infrared (FTIR) spectroscopy measurements were performed with a vacuum Vertex 80v spectrometer in the reflectance mode in the spectral range 1330–25,000 nm at room temperature. The reflectance spectra were converted to absorptance according to the formula *A*_FTIR_(λ,*T*_0_) = 1 – *R*_FTIR_(λ,*T*_0_) and, in turn, the spectral emissivity was retrieved by Kirchhoff’s law of thermal radiation: ε_λ_(λ,*T*_0_) = *A*_FTIR_(λ,*T*_0_).

The Raman spectra were measured on a DXR Raman spectrometer (Thermo Scientific, USA) with a laser operating at 633 nm. The sample was deposited on a silicon wafer (1 × 1 cm), and an excitation laser was focused on its surface. The laser power on the sample was set to 2 mW, and the exposition time was 2 s. Each measured Raman spectrum was an average of 64 experimental scans. The Raman spectra were processed using control software (Omnic, version 8, Thermo Scientific, USA).

X-ray diffraction (XRD) patterns were recorded at room temperature with an Empyrean (PANalytical, The Netherlands) diffractometer in the grazing-incidence mode (GIXRD) geometry, Co Kα radiation (40 kV, 30 mA, λ = 0.1789 nm) equipped with a PIXcel3D detector, and programmable divergence and diffracted beam anti-scatter slits. The samples were placed on a zero-background Si slide. The measurement range was 2θ: 20–80°, with a step size of 0.026°. The GIXRD experiments were performed using an incidence angle of 15°.

X-ray photoelectron spectroscopy (XPS) analysis were performed on a PHI 5000 VersaProbe II XPS system (Physical Electronics) with a monochromatic Al Kα source (15 kV, 50 W) and a photon energy of 1486.7 eV. High-resolution (HR) spectra were scaled using the adventitious carbon peak as a reference at 284.8 eV and fitted with MultiPak (Ulvac-PHI, Inc.) software.

The low-resolution imaging of the catalyst morphology was obtained with a transmission electron microscope (TEM) JEOL equipped with a LaB_6_ emission gun and operating at 160 kV. HR micrographs and STEM elemental mapping were acquired using a FEI Titan HR-TEM microscope equipped with X-FEG electron gun operating at 80 kV.

Scanning electron microscopy (SEM) was used to examine the morphology of the GDEs before and after irradiation. The measurements were carried out with a Scios 2 DualBeam microscope (ultra-HRSEM/FIB, Thermo Fisher Scientific) with an accelerating voltage of 5 kV.

The UV–vis absorption spectra were acquired with a Specord250 Plus spectrometer equipped with an integrating sphere (Analytik Jena GmbH).

The contact angle measurements were carried out using the sessile drop method. A water droplet of 4 μL was placed over the film electrodes, and the image of the droplet was taken by a HR camera. The contact angle was then determined by Image software.

### Electrochemical Measurements

Linear sweep voltammetry (LSV) measurements for Toray paper-based electrodes were carried out in a homemade three-electrode system with a bipotentiostat (Autolab Instruments). The three electrode arrangement consisted of a Pt sheet as the counter electrode (CE), Ag/AgCl (3 M) as the reference electrode (RE), and a working electrode (WE) made by the investigated samples with active geometric area of 0.5 × 1.5 cm on both sides. After 30 min bubbling of O_2_ gas, the WE was inserted into the electrolyte. The testing was conducted after WE was placed into the electrolyte, and cyclic voltammetry (CV) and LSV scans were carried out. The O_2_ was continuously bubbled during the electrochemistry measurements.

Bulk H_2_O_2_ production with Toray paper-based electrodes was carried out in a custom-made two-compartment H-cell with Nafion 117 membrane as the separator. Both the cathode and anode compartment were filled with 30 mL of 0.1 M KOH (pH = 13). H_2_O_2_ production was conducted by chronoamperometry at selected potential with bubbling O_2_ into the cathodic part and magnetic stirring at 900 rpm. The liquid collected at a certain time from the cathodic compartment was analyzed using a commercial test KIT (Merck KGaA, Germany) to quantify H_2_O_2_. In detail, 2 mL of the electrolyte was taken from the cathodic compartment, then diluted with 8 mL of H_2_O, and neutralized by 0.1 M H_2_SO_4_ to achieve the pH between 6 and 8. After that, commercial H_2_O_2_ test solutions were added rapidly to the solution above. After 10 min (reaction time) at room temperature, the color of the resultant mixture solution changed from colorless to orange as hydrogen peroxide reduced copper(II) ions to copper(I) ions in the presence of a phenanthroline derivative. Meanwhile, 2 mL of fresh electrolyte was added back to the cathode compartment to maintain electrolyte volume at 30 mL. Finally, the resultant mixture solution was filled into a standard 3.5 mL cuvette for UV–vis absorption spectra measurements. The absorbance value of the peak at 450 nm was recorded. The concentration–absorbance curves were calibrated using standard hydrogen peroxide solutions, including 0.016, 0.036, 0.055, and 0.076 mmol L^–1^ of H_2_O_2_.

Self-supported TiN/NF@Toray-irr GDEs were assembled in a single-compartment cell for electrochemistry studies such as CVs and LSVs and further in a two-compartment separated H-cell for H_2_O_2_ production and stability investigation. In the assembling cell, TiN/NF@Toray electrodes were in contact with the electrolyte (0.1 M KOH), and the GDE layer was in contact with air, which serves as O_2_ supplier media. Subsequently, the CE was placed in the electrolyte section. The two electrodes were placed on opposite sides closely positioned to theAg/AgCl RE. Two 0.5 cm thick PTFE sheets with 0.2 cm wide by 0.8 cm long square sandwiched the catalyst layer, which served as interface with the liquid electrolyte. The geometric surface area of the catalyst was 0.16 cm^2^.

Mechanistic insights were gained by carrying out electrochemical experiments in a conventional three-electrode system on various types of TiN NPs using a glassy carbon rotating disk electrode (RDE) and a rotating ring-disk electrode (RRDE) including a glassy carbon disk and a Pt ring as the second WE. All potentials were referred to the reversible hydrogen electrode (RHE). The catalyst ink was prepared by suspending the catalyst powder in a mixture containing Milli-Q water and Nafion solution (5 wt %, Sigma-Aldrich). After sonication, the catalyst ink was drop-casted onto the freshly polished glassy carbon electrode and dried at room temperature with humidity control.

RDEs (and RRDEs) were prepared by depositing 5 μL (10 μL) of ink with formulation catalyst/Nafion solution/H_2_O = 3 mg/60 μL/940 μL. For preparing TiN-irr, the catalyst was peeled off from the surface of the TiN/NF@Toray-irr sample by sonication in H_2_O and then used for the formulation of the ink. The potential was scanned between 0 and 1.2 V at 5 mV s^–1^. Before LSV measurements, several CV sweeps from (1.2 to 0 V) at 20 mV s^–1^ were performed to stabilize the catalyst (usually, three cycles until a stable curve was achieved). Koutecky–Levich (K–L) plots were analyzed to calculate the number of electrons (*n*) transferred based on the K–L equation
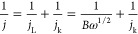

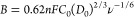
where *j*_k_ is the kinetic current density, *j* is the measured current density, *j*_L_ is the diffusion limiting current density, ω is the angular frequency of rotation (rad s^–1^), *F* is the Faraday constant (96,485 C mol^–1^), *C*_0_ is the bulk concentration of O_2_ (1.26 × 10^–3^ mol L^–1^), *D*_0_ is the diffusion coefficient of O_2_ in 0.1 M H_2_SO_4_ electrolyte (1.93 × 10^–5^ cm^2^ s^–1^), and the kinematic viscosity ν is 0.8926 × 10^–2^ cm^2^ s^–1^ (at 25 °C).^68−71^

During RRDE measurements, the disk potential was scanned between 0 and 0.9 V vs RHE at a scan rate of 5 mV s^–1^ while the ring potential was fixed at 1.2 V vs RHE. The collection efficiency (*N* = 25.0%) of the RRDE was determined by using the [Fe(CN)_6_]^3–^/[Fe(CN)_6_]^4–^ system. H_2_O_2_ selectivity was calculated as follows
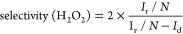
where *I*_d_ is the current detected at the disk and *I*_r_ is the current detected at the ring.

## Results and Discussion

### Fabrication and Characterization of Hybrid Electrocatalysts

**Figure 1:**
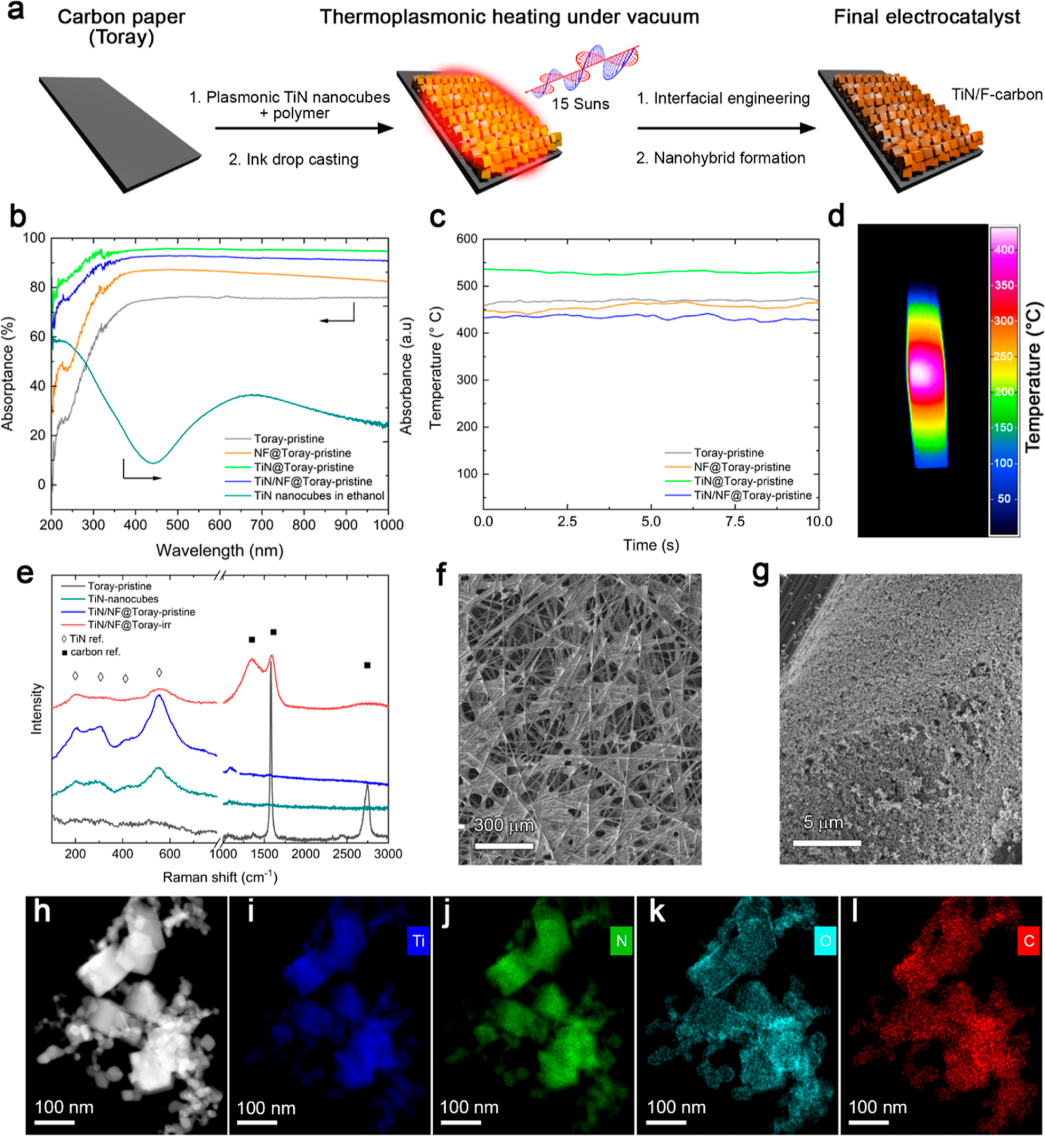
(a) Schematic of the thermoplasmonic fabrication strategy employed for the preparation of TiN/carbon nanohybrid electrodes. (b) Absorption spectra of TiN nanocubes in ethanol (dark green), Toray (gray), NF@Toray (yellow), TiN@Toray (green), and TiN/NF@Toray (blue) electrodes before irradiation. (c) Time-dependent steady-state temperatures under 15 suns irradiation of different samples measured using a thermal camera. (d) Thermal camera image of TiN/NF@Toray under 15 suns irradiation. (e) Raman spectra of Toray paper (black), TiN nanocubes (dark green), TiN/NF@Toray-pristine (blue), and TiN/NF@Toray-irr (red). (f,g) SEM images of TiN/NF@Toray-irr. (h) HAADF STEM micrograph and (i–l) EDS elemental mapping images of the electrocatalyst scratched from the top layer of TiN/NF@Toray-irr.

Mixing inorganic nanocrystals with polymers is an effective strategy to self-assemble them on a substrate forming very efficient broadband solar absorbers.^72^ With this aim, we started the fabrication of our electrodes by forming inks containing plasmonic TiN nanocubes with 30 nm average size (Figure S2), and different ionomer binders dispersed in ethanol showing a localized surface plasmon peak at 650 nm (Figure S3). The synthetic procedure is schematically summarized in Figure 1a. Their subsequent drop-casting onto a porous carbon layer (i.e., Toray paper) produced TiN/binder nanocomposites absorbing above 90% of the incident light in the whole visible range (Figure 1b). To assess the generated photothermal heating (i.e., the thermoplasmonic effect), we excited samples at normal incidence, and an infrared (IR) camera was placed on the back of the films to detect temperature variations. Before acquiring the surface temperature, the spectral emissivity of the synthesized films was determined (Figure S4). The films containing plasmonic TiN nanocubes (TiN@Toray and TiN/NF@Toray) exhibited the highest optical absorption properties in the 300–1100 nm range (Figure 1b) and high photo-induced temperatures (Figure 1c) under 15 suns irradiation. The IR camera image of TiN/NF@Toray shows that there is a gradient of temperatures within the illuminated area, as the center reached 430 °C, while the lowest temperature of ∼300 °C was detected at the periphery of the sample (Figure 1d). These conditions ensured the polymeric binder carbonization under vacuum. After photoirradiation for 2 h under vacuum, the as-synthesized hybrid films were obtained and named TiN/NF@Toray-irr. In contrast, the film before photoirradiation was named TiN/NF@Toray-pristine. A similar labeling was adopted for the films obtained with different organic binders.

The GIXRD patterns of the as-prepared films of the uncoated Toray paper and of the pristine TiN powder are shown in Figure S5. Pure Toray paper shows only one strong peak at 2θ = 30.7° corresponding to the peak of pure graphite,^73,74^ while both TiN/NF@Toray-pristine and Toray-irr show strong signal belonging to the Toray paper and the characteristic diffraction peaks of TiN.^75,76^

The Raman spectrum (Figure 1e) of TiN/NF@Toray-irr shows two broad peaks centered at 1350 and 1585 cm^–1^, which can be attributed to the D and G bands, respectively, both bands being absent in TiN/NF@Toray-pristine. The D band is associated with the sp^3^ C bonds and defects, whereas the G band is related to sp^2^ graphitic carbon.^77,78^ Notably, this spectrum is significantly different from the one of Toray paper, which shows only a sharp peak at 1580 cm^–1^. The additional peaks at ∼423, 302, and 203 cm^–1^ and an intense peak at ∼554 cm^–1^ were assigned to the crystalline TiN phase.^79−81^ All these observations suggest that the thermoplasmonic heating induced the formation of new nanocarbon species deriving from the thermal decomposition of the organic binder, thus producing the formation of hybrid inorganic/organic nanocomposites.

The SEM images of TiN/NF@Toray-irr appear similar to the ones of the pristine sample and confirmed that the carbon fibers forming Toray paper are homogeneously covered with layers of TiN nanocubes self-assembled around them (Figures 1f,g and S6). The film made by closely packed TiN nanocubes can support multiple plasmonic resonance modes and produce electromagnetic hot spots in their contact areas that enable intense local heating and hot carrier generation.^82,83^

**Figure 2:**
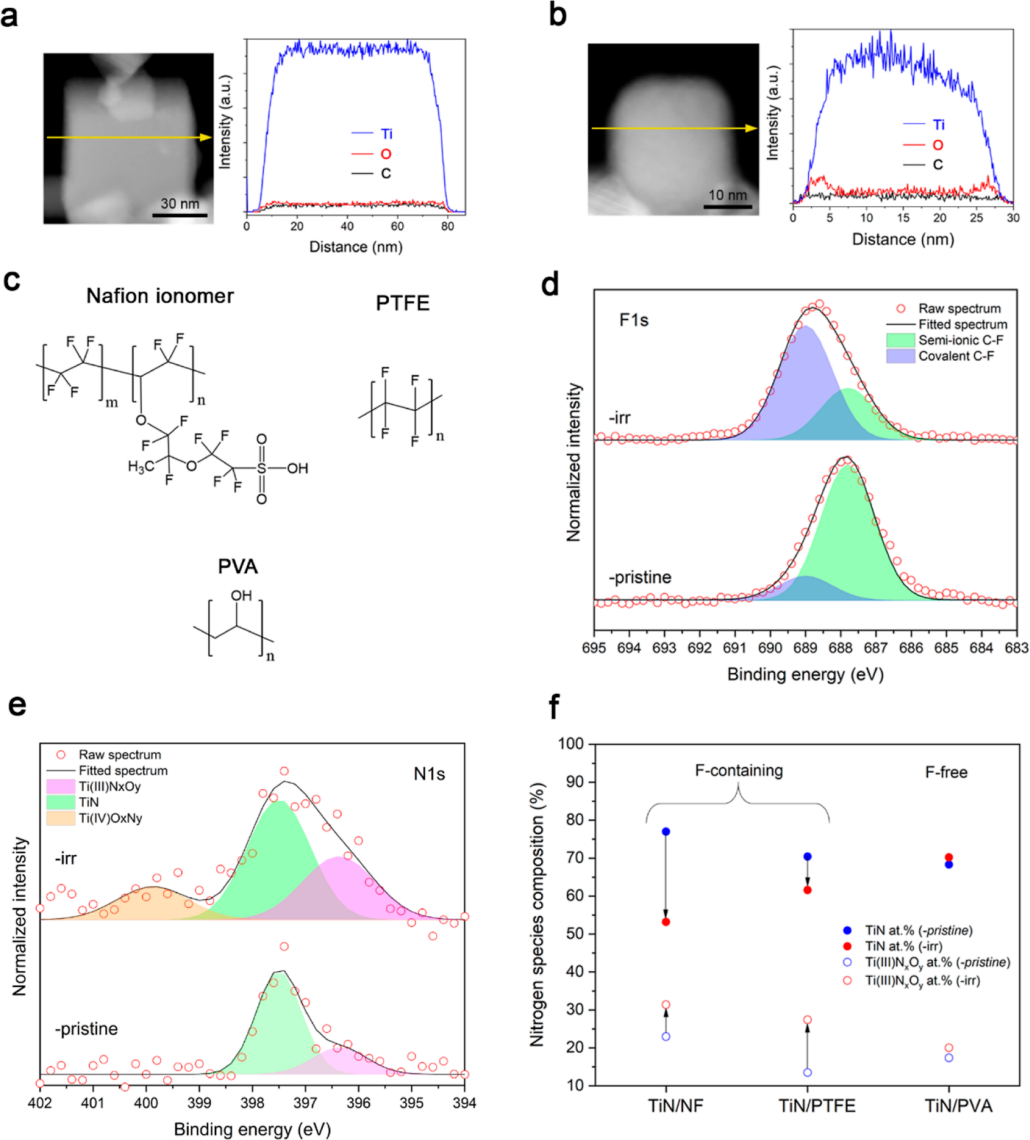
HAADF-STEM micrographs and EDS line scans of single TiN nanocubes from (a) TiN/NF@Toray-pristine and (b) TiN/NF@Toray-irr. (c) Chemical structure of the different polymeric binders used in the nanohybrids. HR-XPS spectra of TiN/NF@Toray-pristine and TiN/NF@Toray-irr in the region of (d) F 1s and (e) N 1s. (f) Comparison of N species content retrieved from the deconvoluted N1s spectra of the investigated samples before and after irradiation.

High-angle annular-dark-field scanning transmission electron microscopy (HAADF-STEM) and energy-dispersive X-ray spectroscopy (EDS) elemental maps of TiN/NF@Toray-irr reveal that Ti, N, and C are evenly distributed at the atomic level, suggesting the formation of TiN/carbon nanohybrids with homogeneous composition upon photoirradiation (Figure 1h–l). Notably, the same analysis performed on TiN nanocubes scraped off from TiN/NF@Toray-pristine shows no significant difference from the irradiated sample neither in particle size and morphology nor in elements distribution, which confirms that photoirradiation did not change the texture of the nanocomposites (Figure S7). The only exception is for the O maps, which evidence a slightly more intense signal for TiN/NF@Toray-irr due to surface oxidation, as typically observed for TiN nanostructures exposed to air.^84^ To better understand this observation, we performed a HAADF-STEM and EDX line scan analysis on single TiN nanocubes belonging both to TiN/NF@Toray-pristine and TiN/NF@Toray-irr (Figure 2a,b). In the latter, the EDS line of O highlights the presence of a thicker layer (∼5 nm) at the surface of the TiN nanocube in comparison with the former, confirming the partial oxidation induced by the organic binder decomposition.

In order to gain insights into the surface chemical composition of the formed nanohybrids, we carried out XPS analysis of all investigated samples containing the following binders: NF, PTFE, and PVA, the latter taken as a reference containing no F species. All the chemical structures are shown in Figure 2c. The surface compositions extracted from the XPS survey spectra (Figure S8 and Table S1) highlight a significant increase of the carbon content and a decrease in Ti for all the films after irradiation. We also observed a significant reduction of both F (NF and PTFE) and O (PVA) content, suggesting the plasmonic-induced decomposition of the original molecular structure along with the in situ formation of new nanocarbons at the TiN surface. In detail, for samples containing NF, the overall relative amount of F species decreased ∼10 at. % after irradiation. The same trend was observed for samples containing PTFE showing 4 at. % decrease of F species, while a stark decrease (∼20 at. %) of O was revealed for TiN/PVA@Toray after irradiation, as expected from the decomposition of hydroxyl groups upon local heating.

The HR-XPS C1s spectra of TiN/NF@Toray-pristine and -irr (Figure S9a and Table S2) can be fitted with six peaks associated to C=C (284.8 eV), C–C (286.0 eV), C–O (287.2 eV), C=O (288.7 eV),^23,85^ C–F (∼289.8 eV), and C–F_2_ (∼291.6 eV).^30,86−88^

Notably, the overall relative amount of oxygenated functional groups increased by 1.5 at. % after irradiation, in agreement with the surface oxidation revealed by HRTEM EDS line scan analysis (Figure 2a). The same trend was observed for samples containing PTFE (Figure S10 and Table S3), while a stark decrease of O was revealed for TiN/PVA@Toray after irradiation, as expected from the decomposition of hydroxyl groups upon local heating (Figure S11a and Table S4).

The HR-XPS F1s spectra of TiN/NF@Toray (Figure 2d and Table S5) and TiN/PTFE@Toray (Figure S10b and Table S6) before and after irradiation could be fitted with two peaks: the first one centered at 688.3 eV was assigned to semi-ionic C–F species, while second one at 689.5 eV belonged to covalent C–F.^30,66,89^ Notably, we observed for both samples after irradiation a drastic concentration decrease of the former and a stark increase of the latter, strongly indicating the plasmonic-induced in situ generation of F-doped nanocarbons.

The formation of fluorinated compounds may induce a change in the wettability of our Toray-based electrodes; therefore, we performed contact angle measurements on selected samples. Interestingly, the contact angle generated by a water droplet with TiN/NF@Toray-pristine and TiN/NF@Toray-irr was 129 and 139°, respectively (Figure S12). These results show a significantly increased hydrophobicity after the thermoplasmonic treatment, suggesting the formation of a superhydrophobic surface as an effect of F-doped nanocarbon formation. Similar contact angles were obtained in the state-of-the-art performing electrode after fluorination and were used to enhance the surface waterproofing performance^90,91^ and improve oxygen transfer.^59,62−64^

To further explore the surface composition variation of the nanocomposites, we now focus the attention to the inorganic component (i.e., the TiN nanocubes).

On the one hand, the HR-XPS Ti2p spectra were deconvoluted using three peaks assigned to TiN, TiN_*x*_O_*y*_, and TiO_2_ species, respectively.^81,84^ The surface analysis of the samples containing fluorinated binders (Figures S9b, S10c and Tables S7, S8) shows a ∼5 at. % increase of the TiO_*x*_ phases (TiN_*x*_O_*y*_ and TiO_2_ species) after illumination, while it remained constant for the samples with PVA (Figure S11b and Table S9).

On the other hand, the HR-XPS N 1s spectra for F-containing samples (Figures 2e and S10d and Tables S10 and S11) were fitted using three peaks, assigned to Ti^(III)^N_*x*_O_*y*_ (∼396.3 eV), TiN (∼397.5 eV), and Ti^(IV)^N_*x*_O_*y*_ (∼399.8 eV), respectively.^39,92−97^Figure 2f reports the relative content of TiN and Ti^(III)^N_*x*_O_*y*_ species retrieved from the spectra of pristine and irradiated samples. Interestingly, the TiN/F-binders (NF and PTFE)@Toray-irr show a 8–14 at.% increase in Ti^(III)^N_*x*_O_*y*_ (corresponding to a 36–100% relative increase) concentration and a concomitant decrease in the TiN content. In contrast, the Ti^(III)^N_*x*_O_*y*_ and TiN content remained nearly constant for TiN/PVA@Toray before and after irradiation (Figure S11c and Table S12). These results highlight that only the plasmonic-induced decomposition of F-containing binders promoted the reconstruction of the TiN nanocubes, favoring the formation of a surface layer richer in Ti^(III)^N_*x*_O_*y*_.

### Role of F-Containing Organic Binders on H_2_O_2_ Electrosynthesis

We first evaluated the performance of the prepared TiN/binder@Toray electrocatalysts in a conventional three-electrode cell. CV scans for TiN/NF@Toray-irr were carried out in Ar- and O_2_-saturated 0.1 M KOH (Figure S13). The enhanced current density observed in the presence of O_2_ demonstrates that the main kinetic current is due to the ORR. The preparation of the TiN/binder@Toray electrocatalysts was optimized in terms of irradiation time (Figure S14) and TiN loading (Figure S15), finding that the highest performance was delivered for 2 h of irradiation using 1 mg of TiN.

**Figure 3:**
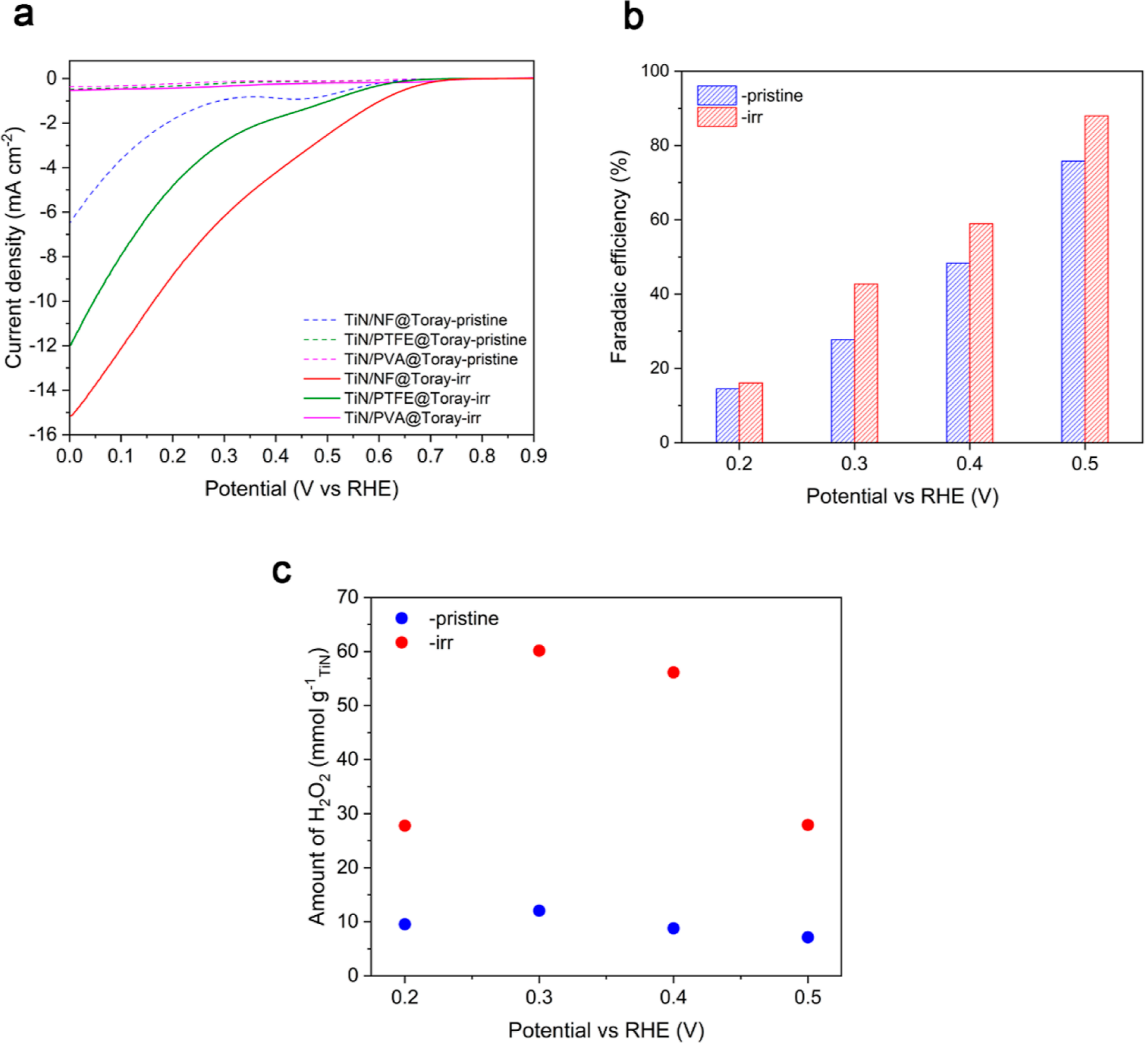
(a) LSV curves of investigated nanohybrid electrodes before and after irradiation obtained in a three-electrode cell. (b) FE and (c) H_2_O_2_ productivity for pristine and irradiated TiN/NF@Toray at different applied potentials using a H-cell configuration. All the measurements were performed in 0.1 M KOH saturated with O_2_.

Figure 3a shows the LSV curves of the investigated electrodes obtained using three different organic binders. Notably, the films containing the fluorinated binders exhibited a remarkable increase in ORR current after being irradiated, while TiN/PVA@Toray showed no ORR activity improvement. For instance, at 0.1 V vs RHE, TiN/PTFE@Toray shows a kinetic current density of 0.42 and 7.86 mA cm^–2^ before and after irradiation, respectively. Similarly, TiN/NF@Toray-irr displays a twice higher performance than TiN/NF@Toray-dark due to the reduced resistance to charge transfer at the solid/liquid interface, as demonstrated by electrochemical impedance spectroscopy measurements (Figure S16).

In order to recognize the contribution of each component forming the nanocomposite electrodes, films made by Toray, NF@Toray, and TiN@Toray were tested before and after irradiation. Figure S17a–c shows that no difference was recorded for neither TiN@Toray nor Toray samples. In contrast, NF@Toray-irr displayed a current density enhancement in comparison to NF@Toray-pristine, suggesting that the photoirradiation also in this case produced fluorinated nanocarbons, which are active electrocatalysts for the ORR.^87,89,98^ Furthermore, this current density comparison highlights that TiN/NF@Toray-irr shows up to 10 times higher current density at relevant applied potentials (see Figure S17d) than the films bearing only the individual components, remarking the crucial role played by the formation of the nanohybrid TiN/F-carbon@Toray electrocatalysts.

To confirm the uniqueness of the plasmon-induced fabrication approach, we prepared a reference TiN/NF@Toray electrocatalyst made by treating it under an inert gas atmosphere at 430 °C in a tubular furnace, replicating the same conditions obtained upon illumination. The LSV of this sample is very similar to the performance of TiN/NF@Toray-pristine (Figure S18), which suggests that both plasmonic local heating and generated hot carriers have a key role for obtaining a functional TiN surface reconstruction and active site formation.

Next, bulk electrolysis with the planar electrode was performed in a custom designed H-cell (Figure S19**)** by recording chronoamperometry curves (Figure S20) of TiN/NF@Toray at different applied potentials and monitoring the formation of H_2_O_2_ by a photometric method (see details in the Experimental Section). Figure 3b shows the calculated FE at four different applied bias for the sample before and after irradiation. The FE toward H_2_O_2_ increases from around 15–20% at 0.2 V vs RHE to 75–90% at 0.5 V vs RHE, where a remarkable FE as high as ∼90% for TiN/NF@Toray-irr was obtained. Notably, TiN/NF@Toray-irr always shows higher FE toward H_2_O_2_ than TiN/NF@Toray-pristine at any of the applied potentials explored. This trend is further highlighted in the H_2_O_2_ productivity plot (Figure 3c), which appears with a volcano shape reaching the maximum at 0.3 V vs RHE where the TiN/NF@Toray-irr reached ∼60 mmol g_TiN_^–1^ h^–1^of H_2_O_2_ productivity, six times more than the pristine electrocatalyst.

### Role of Ti^(III)^N_*x*_O_*y*_ on the H_2_O_2_ Selectivity

To clarify how the surface Ti species influence the selectivity toward H_2_O_2_ formation, we performed RDE and RRDE experiments with electrocatalysts containing varying surface compositions. We compare pristine TiN nanocubes, TiN nanocubes treated at 800 °C for 2 h under ammonia (TiN–NH_3_), and the powder scraped from TiN/NF@Toray-irr. The high-temperature treatment did not change the crystalline structure of TiN nanocubes (Figure S21) but allowed us to tune their surface compositions. In this way, we obtained a series of electrocatalysts featuring an increasing surface content of TiN (TiN–NH_3_ > TiN nanocubes > TiN/NF@Toray-irr) and a concomitant decrease of Ti^(III)^N_*x*_O_*y*_ concentration (TiN–NH_3_ < TiN nanocubes < TiN/NF@Toray-irr), as retrieved from XPS measurements (Figure S22, Tables S13 and S14, referring to TiN/NF@Toray-irr Figures S9b, 2e and Tables S7 and S10).

From the LSV curves acquired using RDEs at different rotating speeds, we obtained K−L plots showing good linearity for all the investigated electrocatalysts (Figures S23–S25). Interestingly, the number of electrons characterizing the electrochemical process (*n*) shows that TiN/NF@Toray-irr (i.e., the sample containing the lowest TiN and highest Ti^(III)^N_*x*_O_*y*_ surface content) catalyzes the 2e^–^ ORR (i.e., production of H_2_O_2_) in a significantly wider potential window (0.2–0.6 V vs RHE) in comparison with the sample having highest TiN and lowest Ti^(III)^N_*x*_O_*y*_ content (TiN–NH_3_) which was active only between 0.5 vs RHE and 0.6 V vs RHE. We also carried out RDE experiments of TiO_2_ NPs showing that the oxide phase is able to drive ORR following the 2e^–^ pathway in the same potential range (0.1–0.6 V vs RHE) but with much lower current density in comparison to TiN due to its insulating properties (Figure S26).

The RDE polarization curves discussed above show, for all samples, the presence of current maxima accompanied by only gradual current increase. This has been more or less confidently assigned by several authors to an ORR that proceeds under diffusion control or at least mixed diffusion-kinetic control regime.^99,100^ The presence of such peaks gave rise to speculations about possible complex mechanistic steps: for example, the peaks could be associated to one-electron O_2_ reduction to O_2_^–^ which in alkaline solution can survive long enough to disproportionate and produce H_2_O^–^;^99,101^ alternatively, mixed 2e^–^ + 2e^–^ and 4e^–^ reduction processes have been hypothesized to lead to the formation of both H_2_O_2_ and H_2_O.^100^

The selectivity trend observed in RDE measurements was confirmed by further electrocatalytic tests using the RRDE, which allowed us to assess electrochemically the H_2_O_2_ selectivity (Figure S27). In detail, TiN/NF@Toray-irr is the most selective electrocatalyst for H_2_O_2_ production, showing a constant selectivity around 80% between 0.6 and 0.3 V vs RHE. Similar performances were recently observed in RRDE configuration for highly active O-and F-doped carbons creating an engineered reaction microenvironment for H_2_O_2_ electrosynthesis.^58^ At lower potentials, the selectivity decreased reaching a still considerable 40% at 0.05 V vs RHE. TiN nanocubes showed a similar potential-dependent selectivity behavior but with lower performance in comparison of TiN/NF@Toray-irr. Otherwise, TiN–NH_3_ shows a very low activity toward H_2_O_2_ synthesis, as evidenced by the 60% selectivity obtained only at 0.6 V vs RHE (where the kinetic current is at minimum values) and very rapidly decreasing to 5–10% already at 0.3 V vs RHE. These results highlight that the electrocatalyst featuring the highest Ti^(III)^N_*x*_O_*y*_ surface content shows the highest H_2_O_2_ selectivity in a wider range of applied potentials. They also suggest that the performances of TiN-hybrid electrocatalysts are ensured on the one hand by the metallic conductivity of TiN (much higher than that of insulating TiO_2_), while on the other hand, the passivated interface formed by Ti^(III)^N_*x*_O_*y*_/TiO_2_/F-nanocarbons is crucial to stir the ORR selectivity toward the 2e^–^ pathway, shifting the activity paradigm for metal nitrides that usually prefer the 4e^–^ mechanism.^27^

### Electrochemical Testing with Natural Air Diffusion

Testing the films in a three-electrode configuration and completely immersing the Toray paper supporting the electrocatalysts in the electrolyte, we noted that their stability was limited, especially running bulk electrolysis at 0.2 V vs RHE of applied bias (i.e., when electrodes work at higher current densities) (Figure S20). This could be due to the flooding of the electrolyte through the Toray paper which produced a partial deactivation of the electrocatalysts.^102^

**Figure 4:**
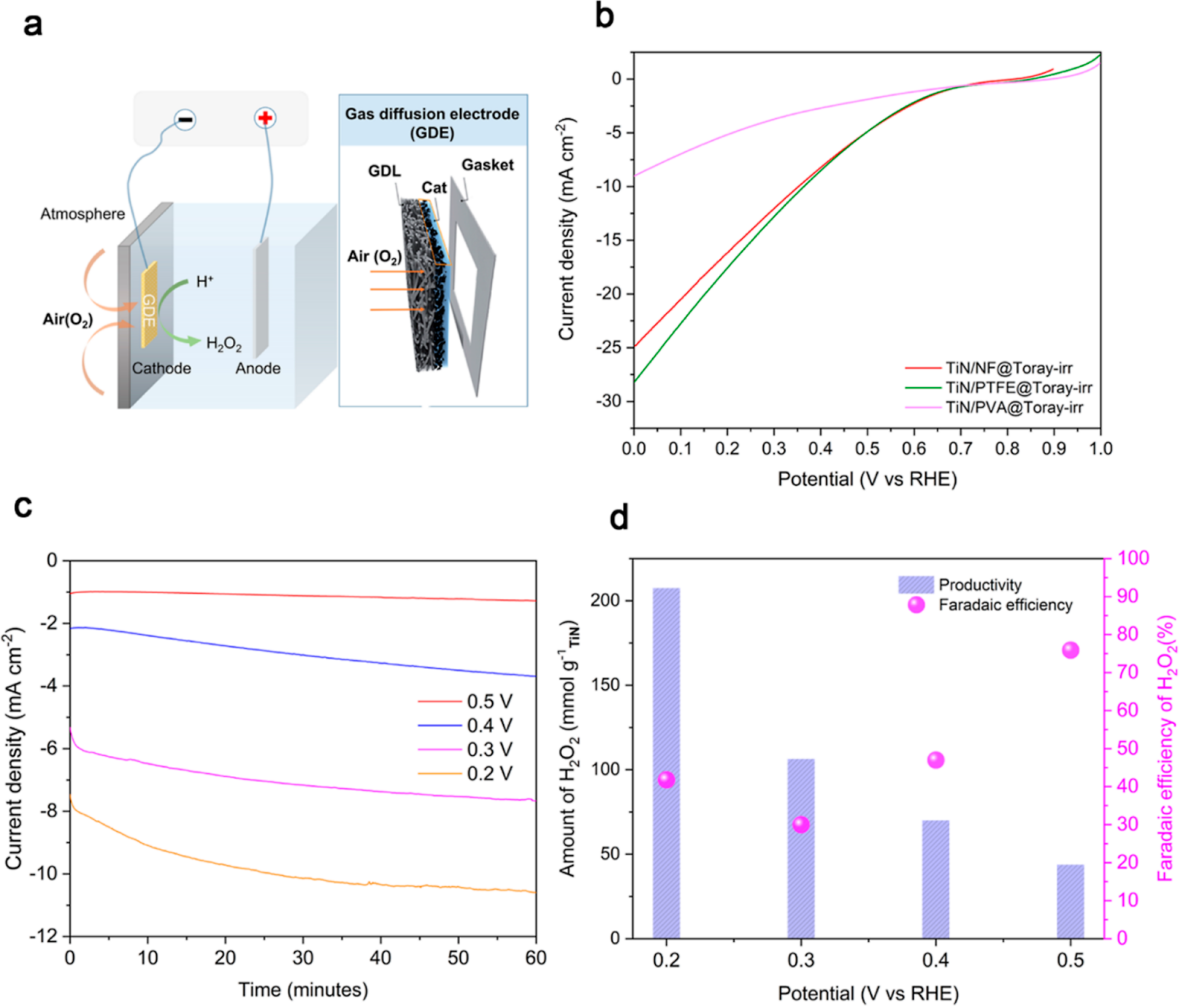
(a) Schematic of the GDE configuration with spontaneous air feeding. (b) Comparison of LSV curves of the electrodes containing the different organic binders after irradiation and tested using GDE configuration using a three-electrode cell in 0.1 M KOH. (c) Electrocatalytic performance of TiN/PTFE@Toray-irr in H-cell with GDEA: (c) chronoamperometry at different applied potentials and (d) corresponding FE and H_2_O_2_ productivity for TiN/PTFE@Toray-irr measured using a H-cell in 0.1 M KOH.

To solve this puzzle, we conducted ORR in a GDE configuration (Figure 4a), allowing natural air diffusion through the GDEs into the electrolyte and thus the spontaneous feeding oxygen to the cathode surface without the need for bubbling it into the electrolyte.

The LSV measurements carried out in this configuration show that the nanocomposites including the fluorinated nanocarbons achieved again the highest ORR current density, with TiN/PTFE@Toray-irr and TiN/NF@Toray-irr reaching ∼25 mA cm^–2^ at 0.1 V vs RHE (Figure 4b), while TiN/PVA@Toray-irr showing a much lower current (∼7 mA cm^–2^ at 0.1 V vs RHE), in agreement with the three-electrode cell tests. Importantly, bulk electrolysis with air GDEs performed in a custom designed H-cell (as shown in Figure S28) highlights the high stability of TiN/PTFE@Toray-irr even when high current density passes through the electrode (Figure 4c). This was further confirmed by the stability cycling test where the LSV curve obtained after 160 sweeps almost overlaps with the one obtained using the fresh electrode (Figure S29).

Notably, the natural air diffusion H-cell measurements with GDE made by TiN/PTFE@Toray-irr enabled to achieve a high H_2_O_2_ selectivity at 0.5 V vs RHE (∼80%) and an increased H_2_O_2_ productivity of 207 mmol g_TiN_^–1^ h^–1^at 0.2 V vs RHE with ∼40% H_2_O_2_ selectivity (Figure 4d). These values are 4–5 folds higher than that obtained in the conventional H-cell (Figure 3c) and could reach state-of-the art performances for carbon-based electrodes by engineering a custom-made flow cell.^58^

## Conclusions

In summary, a TiN/F-doped nanocarbon hybrid was in situ prepared over GDE by local plasmonic heating. The electrode exhibited high stability, activity, and selectivity toward H_2_O_2_ formation during the ORR. Superior catalytic performances were achieved only when using F-containing polymers as binders, implying that the fluorination modification by both plasmonic heating and hot carriers played a crucial role in enhancing the activity of the nanohybrids. Mechanistic studies indicated that a surface enrichment in Ti^(III)^N_*x*_O_*y*_ species obtained upon thermoplasmonic treatment determined an enhanced H_2_O_2_ selectivity. The in situ formed F-doped nanocarbons also provided superhydrophobic surface properties, thus enhancing the stability of the electrodes during operation at high current densities. Our methodology opens the way to large-scale manufacturing of superhydrophobic GDEs by surface plasmonic heating and can be implemented for a variety of energy- and industrially relevant electrochemical processes.

## Supporting Information Available

Characterizations of materials and electrodes and electrochemical tests (PDF)
