# Supplementary material for: Thermoplasmonic In Situ Fabrication of Nanohybrid Electrocatalysts over Gas Diffusion Electrodes for Enhanced H2O2 Electrosynthesis
Source: ACS Catal. 2023 Jul 20;13(15):10205–16. doi: 10.1021/acscatal.3c01837 (PMC10407842; doi:10.1021/acscatal.3c01837)
Supplement: Supplementary file 1 — cs3c01837_si_001.pdf [file cs3c01837_si_001.pdf]

Supporting Information

Thermoplasmonic in situ fabrication of nanohybrid electrocatalysts over gas diffusion electrodes for enhanced H_2O_2 electrosynthesis

Yu Zhang,[†] *Luca Mascaretti,*[†] *Michele Melchionna,*[#] *Olivier Henrotte,*[†] *Štepan Kment,*[†,≠] *Paolo Fornasiero,*[#] *Alberto Naldoni*[‡]*

[†] Czech Advanced Technology and Research Institute, Regional Centre of Advanced Technologies and Materials, Palacký University Olomouc, Šlechtitelů 27, 78371Olomouc, Czech Republic

[#] Department of Chemical and Pharmaceutical Sciences, ICCOM-CNR Trieste Research Unit, INSTM-Trieste, Center for Energy, Environment and Transport Giacomo Ciamician, University of Trieste, Via L. Giorgieri 1, 34127 Trieste, Italy

[≠] Nanotechnology Centre, Centre of Energy and Environmental Technologies, VŠB–Technical University of Ostrava, 17. listopadu 2172/15, Poruba, 708 00 Ostrava, Czech Republic

[‡] Department of Chemistry and NIS Centre, University of Turin, 10125Turin, Italy

Email: alberto.naldoni@unito.it

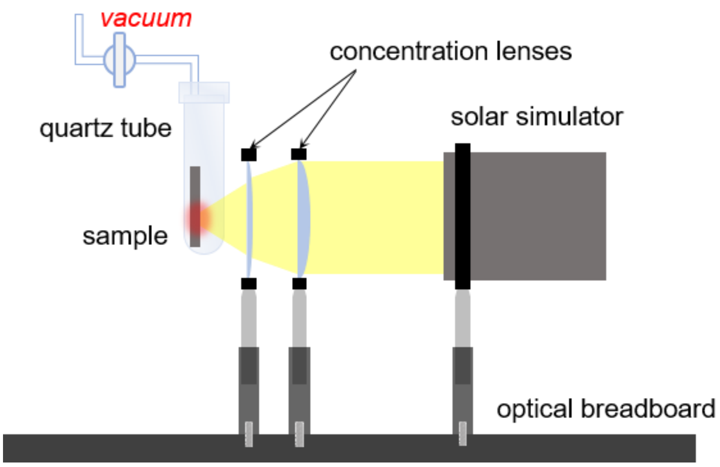

Figure S1. Schematic of photo-irradiation process under concentrated solar-simulated light to employed for the thermoplasmonic synthesis of TiN/NF hybrids on Toray paper. The same process was applied also to other control samples.

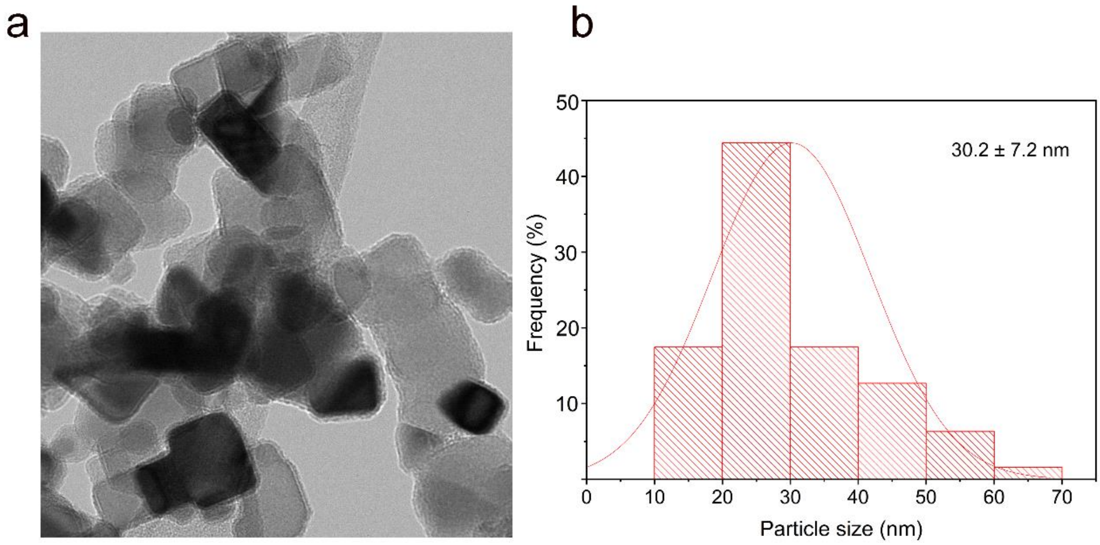

Figure S2. Morphology characterizations of commercial TiN nanocubes: a) selected area representative transmission electron microscopy (TEM) image and b) corresponding particle size distribution.

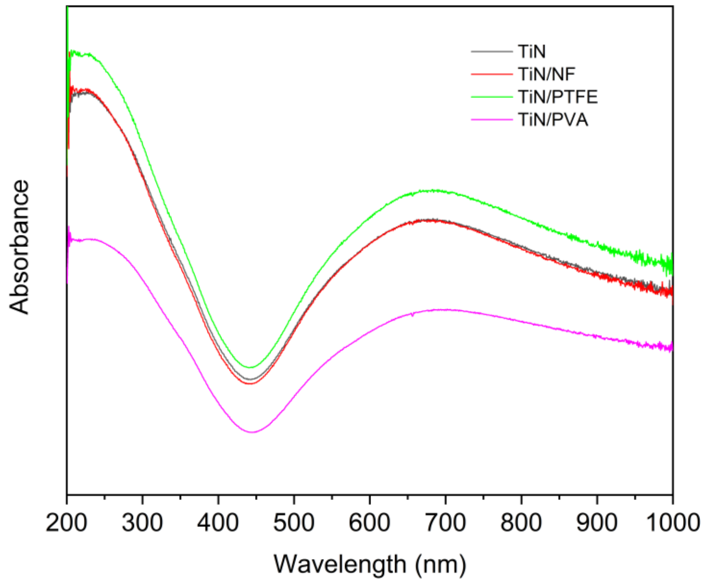

Figure S3. UV–Vis absorption spectra of commercial TiN nanocubes after be prepared as the ink with different binders: Nafion (TiN/NF), polytetrafluoroethylene (TiN/PTFE), poly-vinyl alcohol (TiN/PVA) and the reference sample commercial TiN nanocubes in ethanol without binders (TiN).

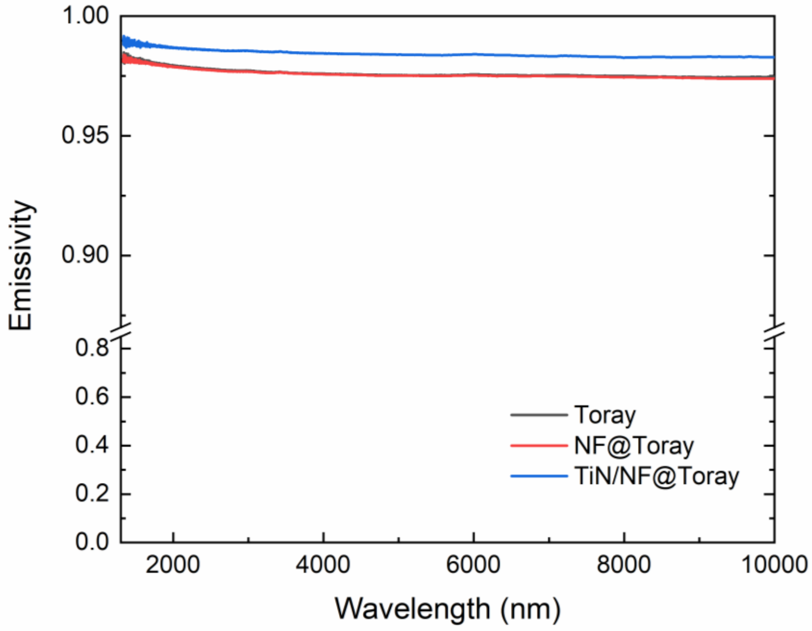

Figure S4. Spectral emissivity for the different electrodes (Toray, NF@Toray and TiN/NF@Toray) retrieved by FTIR spectroscopy in vacuum at room temperature.

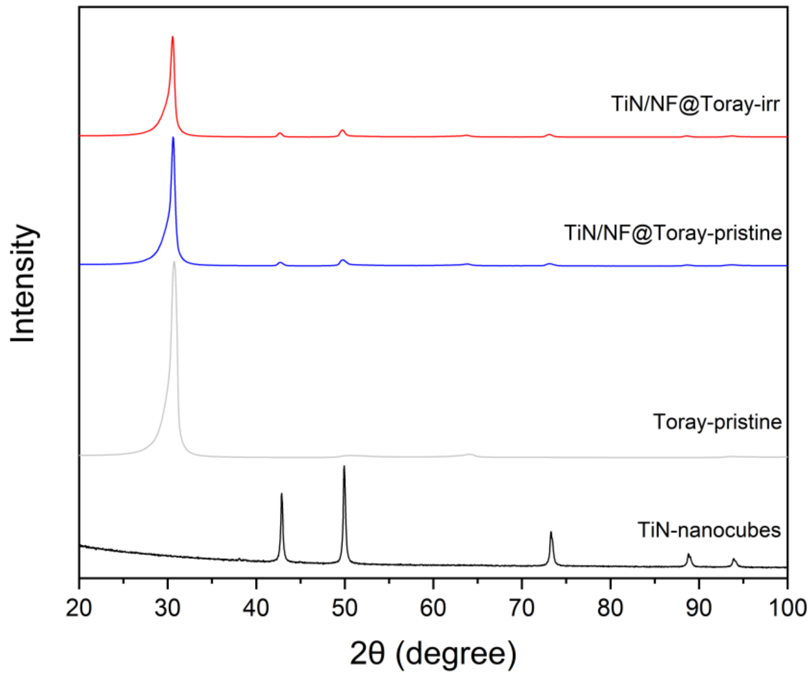

Figure S5. XRD patterns of TiN/NF@Toray samples before (-pristine) and after photo-irradiation (-irr), where pure Toray carbon paper without photo-irradiation (Toray-pristine) and commercial TiN nanocuebs (TiN-nanocubes) were also collected for references.

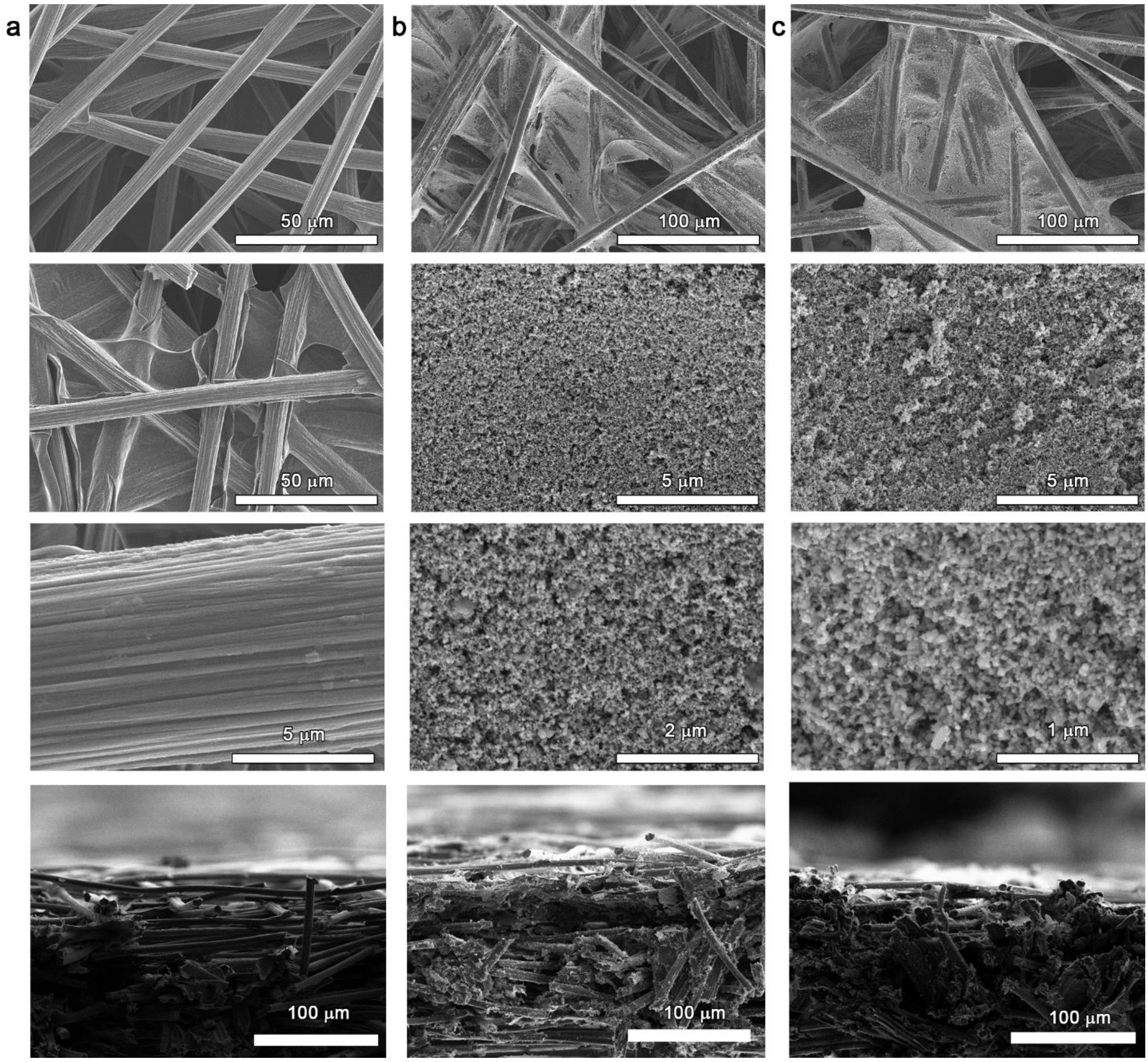

Figure S6. SEM images include cross-section images of a) pristine Toray paper, b) before photo-irradiation (TiN/NF@Toray-pristine), and c) TiN/NF@Toray samples after photo-irradiation (TiN/NF@Toray-irr).

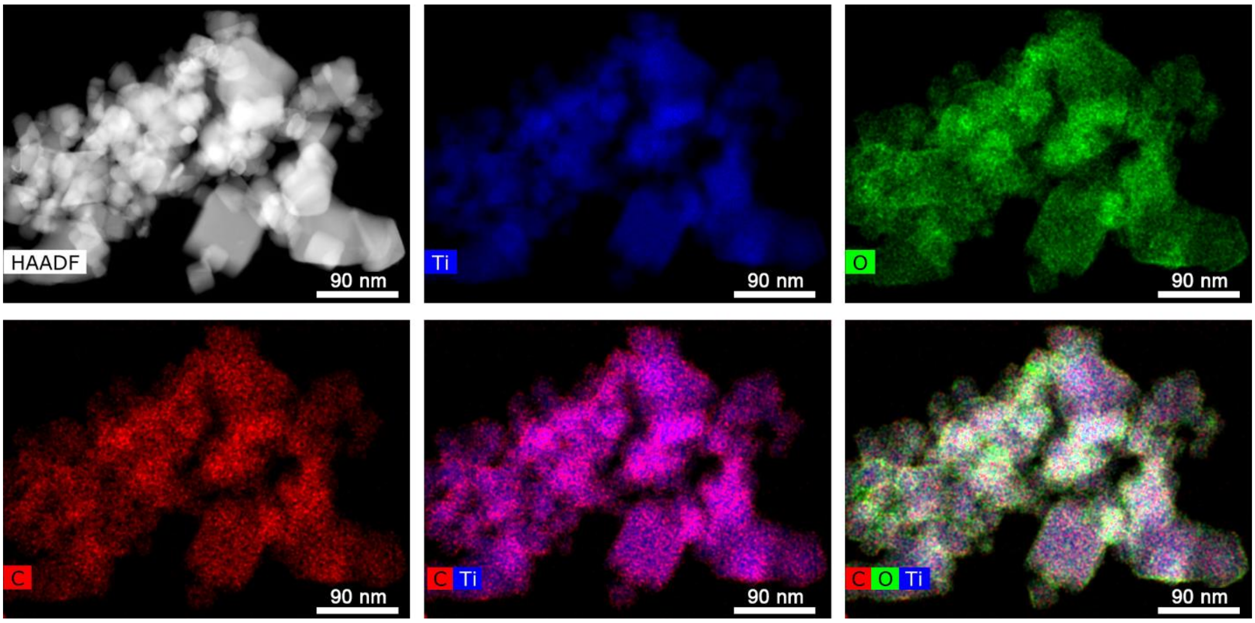

Figure S7. High angle annular dark-field (HAADF) images and energy-dispersive X-ray spectroscopy (EDS) elemental mappings HR-TEM analysis of the TiN particles scraped from the top layer of TiN/NF@Toray sample before photo-irradiation (TiN/NF@Toray-pristine).

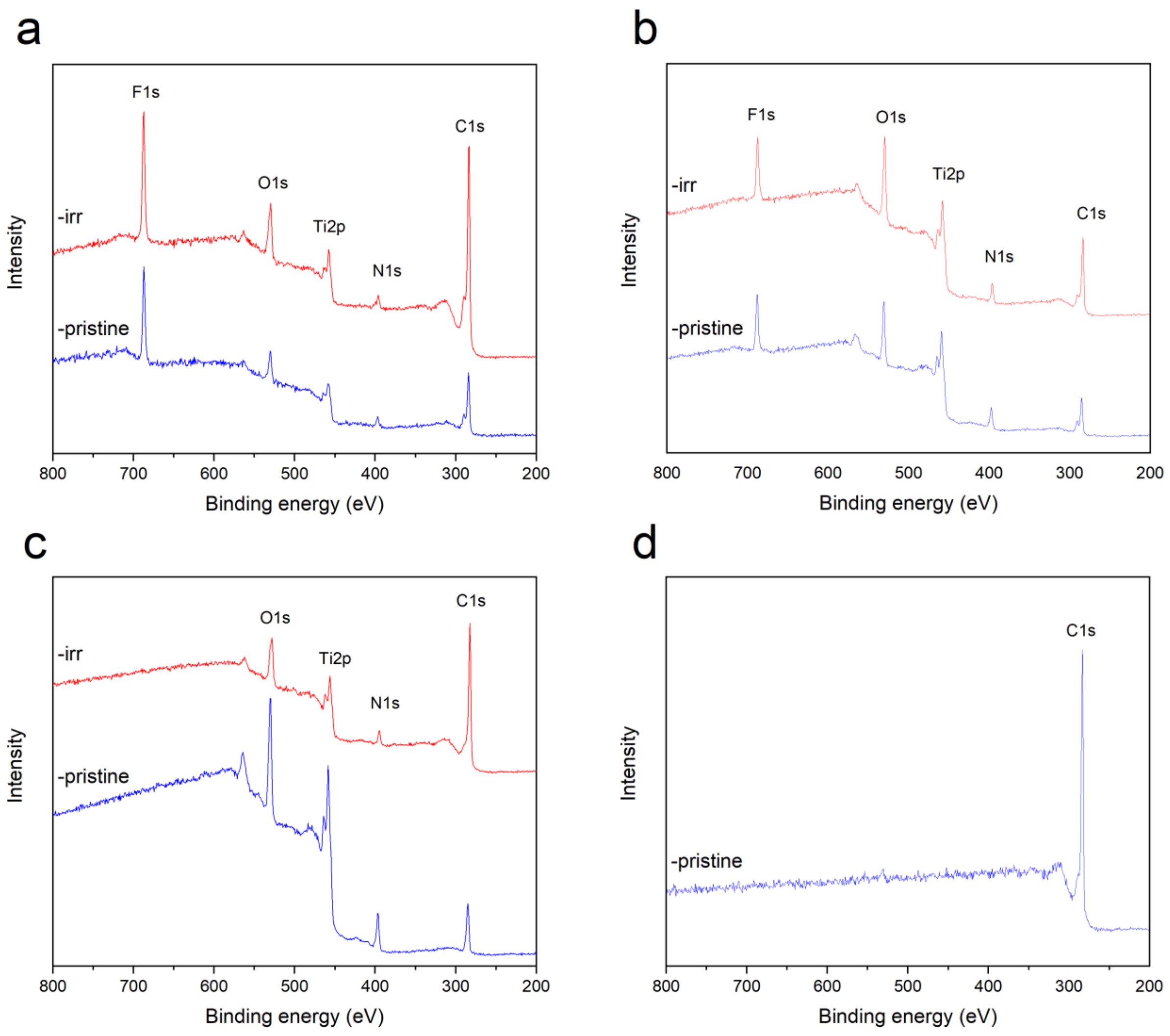

Figure S8. Survey XPS spectra analysis for TiN drop-casted Toray film electrode with different binders before (-pristine) and after photo-irradiation (-irr): a) TiN/NF@Toray, b) TiN/PTFE@Toray, c) TiN/PVA@Toray, and d) Toray carbon substrate before photo-irradiation (Toray-pristine).

Table S1. Chemical composition analysis of TiN/binder@Toray before (-pristine) and after photo-irradiation (-irr), according to the survey XPS spectra. Pristine Toray paper was used as the reference.

Entry	***Sample***	***Ti (at. %)***	***N (at.%)***	***O (at. %)***	***C (at. %)***	***F (at. %)***
**1**	**TiN/NF@Toray-pristine**	6.0	4.9	12.6	53.8	22.8
**2**	**TiN/NF@Toray-irr**	1.5	3.1	10.5	72.1	12.8
**3**	**TiN/PTFE@Toray-pristine**	14.5	9.3	21.2	40.3	14.7
**4**	**TiN/PTFE@Toray-irr**	10.3	5.8	23.1	50.1	10.7
**5**	**TiN/PVA@Toray-pristine**	20.6	13.9	34.8	30.7	-
**6**	**TiN/PVA@Toray-irr**	6.2	4.4	13.4	76.0	-
**7**	**Toray-pristine**	98.0	-	2.0	-	-

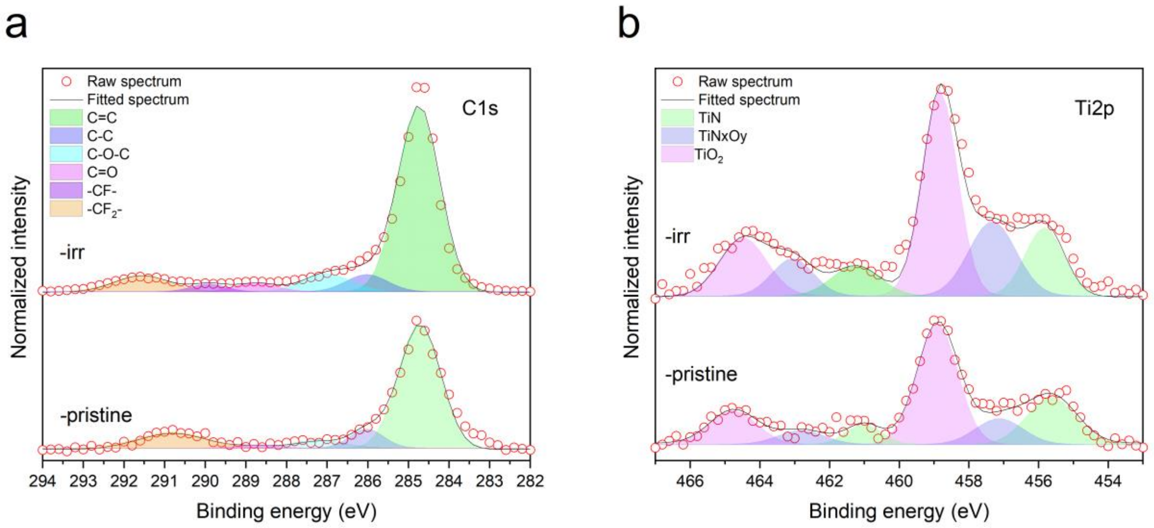

Figure S9. HR-XPS spectra of TiN/NF@Toray-pristine and TiN/NF@Toray-irr in the region of a) C 1s, and b) Ti2p.

Table S2. The relative amount of the different C species according to the deconvolution of HR XPS spectra of C1s for TiN/NF@Toray before (-pristine) and after photo-irradiation (-irr).

C species	*TiN/NF@Toray-pristine*	*TiN/NF@Toray-irr*
**C=C (at.%)**	69.4	71
**C-C (at.%)**	8.7	6.6
**C-O (at.%)**	5.3	5.1
**C=O (at.%)**	1.7	3.4
**-C-F- (at.%)**	-	2.5
**$-CF_2-$ (at.%)**	14.9	8.4

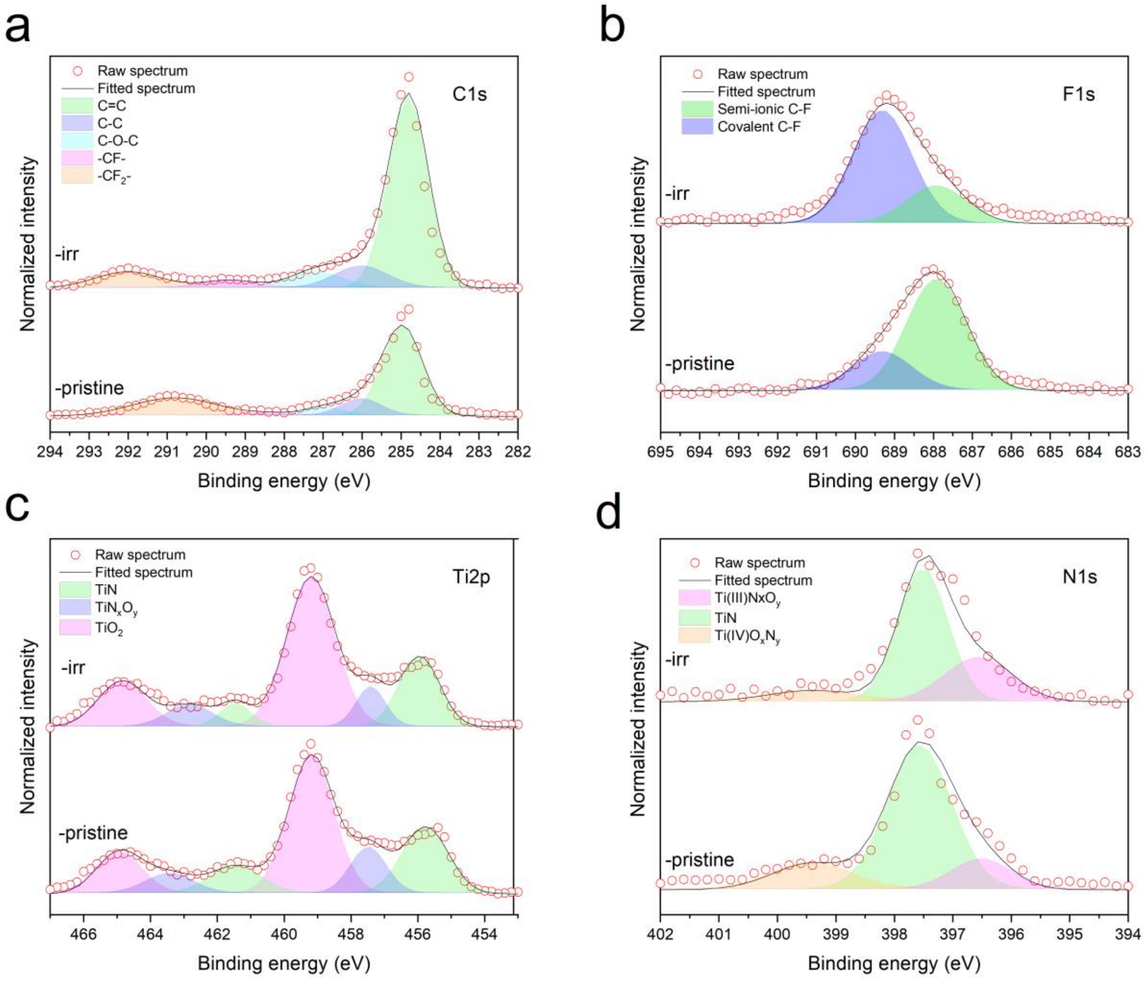

Figure S10. HR-XPS spectra of TiN/PTFE@Toray-pristine and TiN/PTFE@Toray-irr in the region of a) F1s, b) C 1s, c) Ti2p, and d) N 1s.

Table S3. The relative amount of the different C species according to the deconvolution of HR XPS spectra of C1s for TiN/PTFE@Toray before (-pristine) and after photo-irradiation (-irr).

C species	*TiN/PTFE@Toray-pristine*	*TiN/PTFE@Toray-irr*
**C=C (at.%)**	56.8	68.0
**C-C (at.%)**	10.6	10.4
**C-O (at.%)**	5.5	7.9
**-C-F- (at.%)**	1.7	3.8
**-CF_2- (at.%)**	25.4	9.9

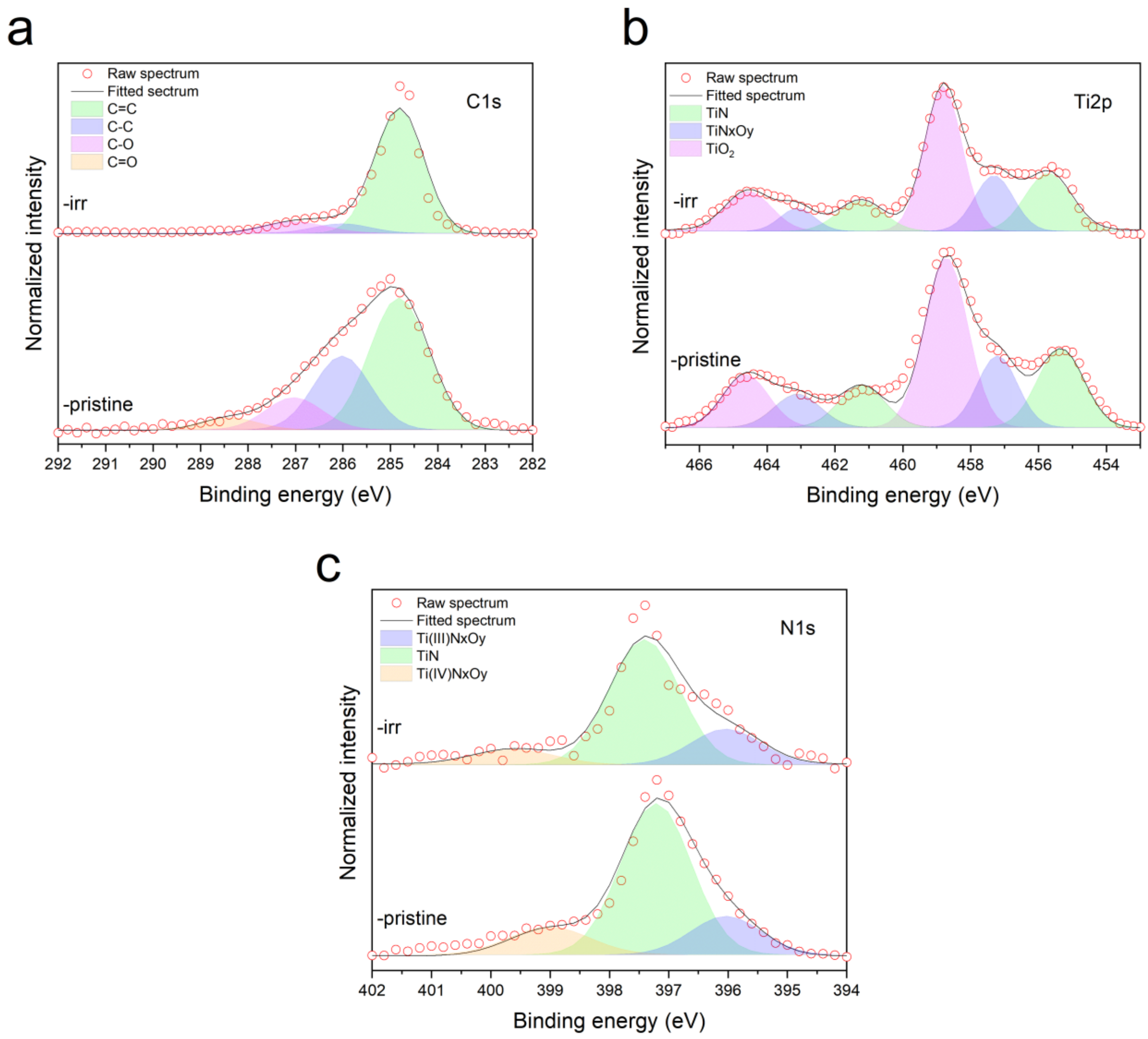

Figure S11. XPS analysis of TiN/PVA@Toray for before (-pristine) and after irradiation (-irr). a) HR XPS spectra of C1s and deconvoluted peaks, b) HR XPS spectra of Ti2p and deconvoluted peaks, and c) HR XPS spectra of N1s and deconvoluted peaks.

Table S4. The relative amount of the different C species according to the deconvolution of HR XPS spectra of C1s for TiN/PVA@Toray before (-pristine) and after photo-irradiation (-irr).

C species	*TiN/PVA@Toray-pristine*	*TiN/PVA@Toray-irr*
**C=C (at.%)**	53.7	82.34
**C-C (at.%)**	28.9	7.3
**C-O (at.%)**	12.6	10.3
**C=O (at.%)**	4.8	-

Table S5. The relative amount of the different F species according to the deconvolution of HR XPS spectra of N1s for TiN/NF@Toray before (-pristine) and after photo-irradiation (-irr).

F species	*TiN/NF@Toray-pristine*	*TiN/NF@Toray-irr*
**Semi ionic C-F (at.%)**	84.9	31.1
**Covalent C-F (at.%)**	15.1	68.9

Table S6. The relative amount of the different F species according to the deconvolution of HR XPS spectra of N1s for TiN/PTFE@Toray before (-pristine) and after photo-irradiation (-irr).

F species	*TiN/PTFE@Toray-pristine*	*TiN/PTFE@Toray-irr*
**Semi ionic C-F (at.%)**	74.4	24.8
**Covalent C-F (at.%)**	25.6	75.2

Table S7. The relative amount of the different Ti species according to the deconvolution of HR XPS spectra of Ti2p for TiN/NF@Toray before (-pristine) and after photo-irradiation (-irr).

Ti species	*TiN/NF@Toray-pristine*	*TiN/NF@Toray-irr*
**TiN (at.%)**	26.3	21.3
**TiN_xO_y (at.%)**	16.4	26.3
**TiO_2 (at.%)**	57.3	52.4

Table S8. The relative amount of the different Ti species according to the deconvolution of HR XPS spectra of Ti2p for TiN/PTFE@Toray before (-pristine) and after photo-irradiation (-irr).

Ti species	*TiN/PTFE@Toray-pristine*	*TiN/PTFE@Toray-irr*
**TiN (at.%)**	26.3	22.9
**TiN_xO_y (at.%)**	17	14.1
**TiO_2 (at.%)**	53.8	63

Table S9. The relative amount of the different Ti species according to the deconvolution of HR XPS spectra of Ti2p for TiN/PVA@Toray before (-pristine) and after photo-irradiation (-irr).

Ti species	*TiN/PVA@Toray-pristine*	*TiN/PVA@Toray-irr*
**TiN (at.%)**	28.3	28.5
**TiOxNy (at.%)**	22.3	20.7
**TiO_2 (at.%)**	49.4	50.8

Table S10. The relative amount of the different N species according to the deconvolution of HR XPS spectra of N1s for TiN/NF@Toray before (-pristine) and after photo-irradiation (-irr).

N species	*TiN/NF@Toray-pristine*	*TiN/NF@Toray-irr*
**TiN (at.%)**	77	53.2
**Ti(III)N_xO_y (at.%)**	23	31.4
**Ti(IV)N_xO_y (at.%)**	-	16.4

Table S11. The relative amount of the different N species according to the deconvolution of HR XPS spectra of N1s for TiN/PTFE@Toray before (-pristine) and after photo-irradiation (-irr).

N species	*TiN/PTFE@Toray-pristine*	*TiN/PTFE@Toray-irr*
**TiN (at.%)**	70.4	61.6
**Ti(III)N_xO_y (at.%)**	13.5	27.4
**Ti(IV)O_xN_y (at.%)**	16.1	11.0

Table S12. The relative amount of the different N species according to the deconvolution of HR XPS spectra of N1s for TiN/PVA@Toray before (-pristine) and after photo-irradiation (-irr).

N species	*TiN/PVA@Toray-pristine*	*TiN/PVA@Toray-irr*
**TiN (at.%)**	68.3	70.2
**Ti(III)NxOy (at.%)**	17.4	20.0
**Ti(IV)NxOy (at.%)**	14.3	9.8

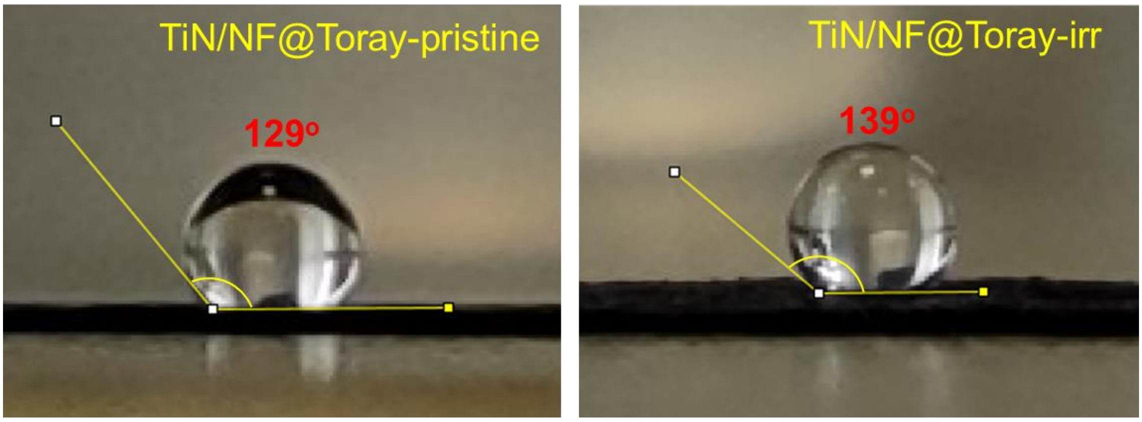

Figure S12. Changes in water contact angle (hydrophobicity) of TiN/NF@Toray before (-pristine) and after photo-irradiation (-irr).

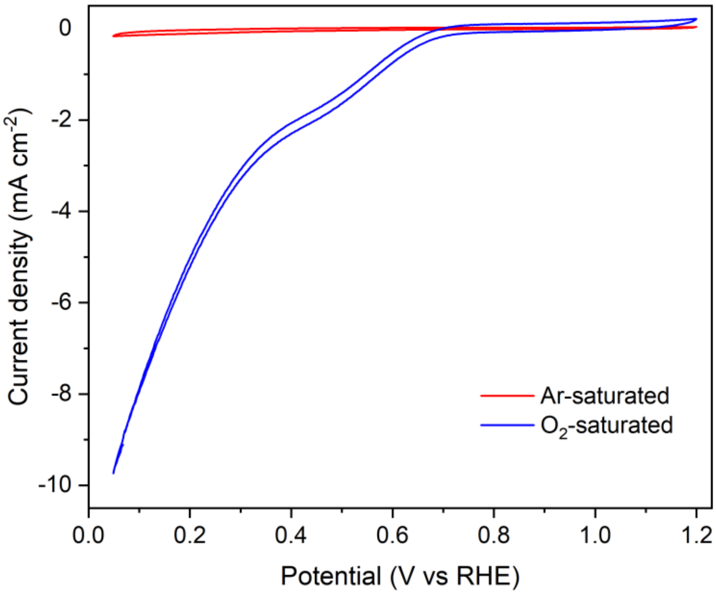

Figure S13. Comparison of cyclic voltammetry curves under Ar (red line)− and O_2 (blue line) −saturated electrolyte solution (0.1 M KOH, pH=13) for TiN/NF@Toray-irr. The curves were collected at a scan rate of 20 mV s^{-1} with a planar film electrode.

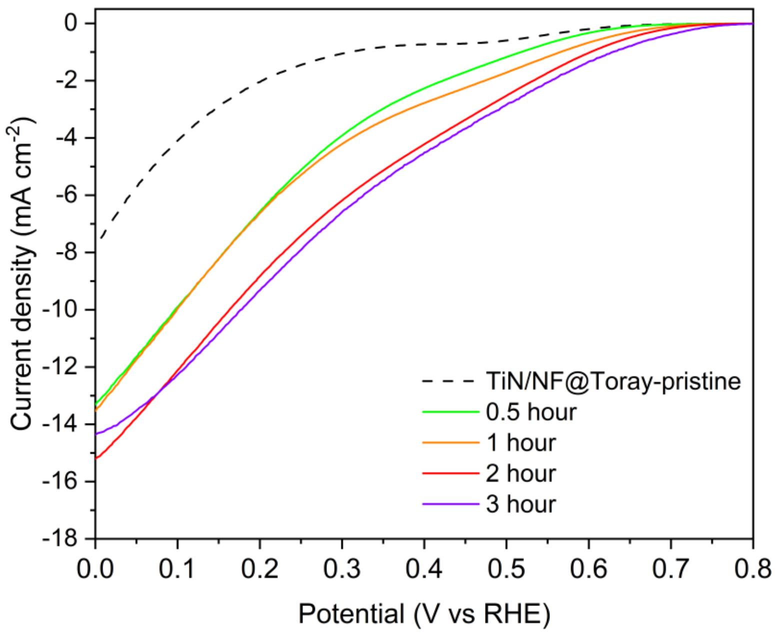

Figure S14. ORR performances of TiN/NF@Toray-irr prepared using different photo-irradiation times.All the measurements were collected in O_2–saturated 0.1 M KOH at a scan rate of 5 mV s^{-1}, where TiN/NF@Toray-pristine is displayed for reference.

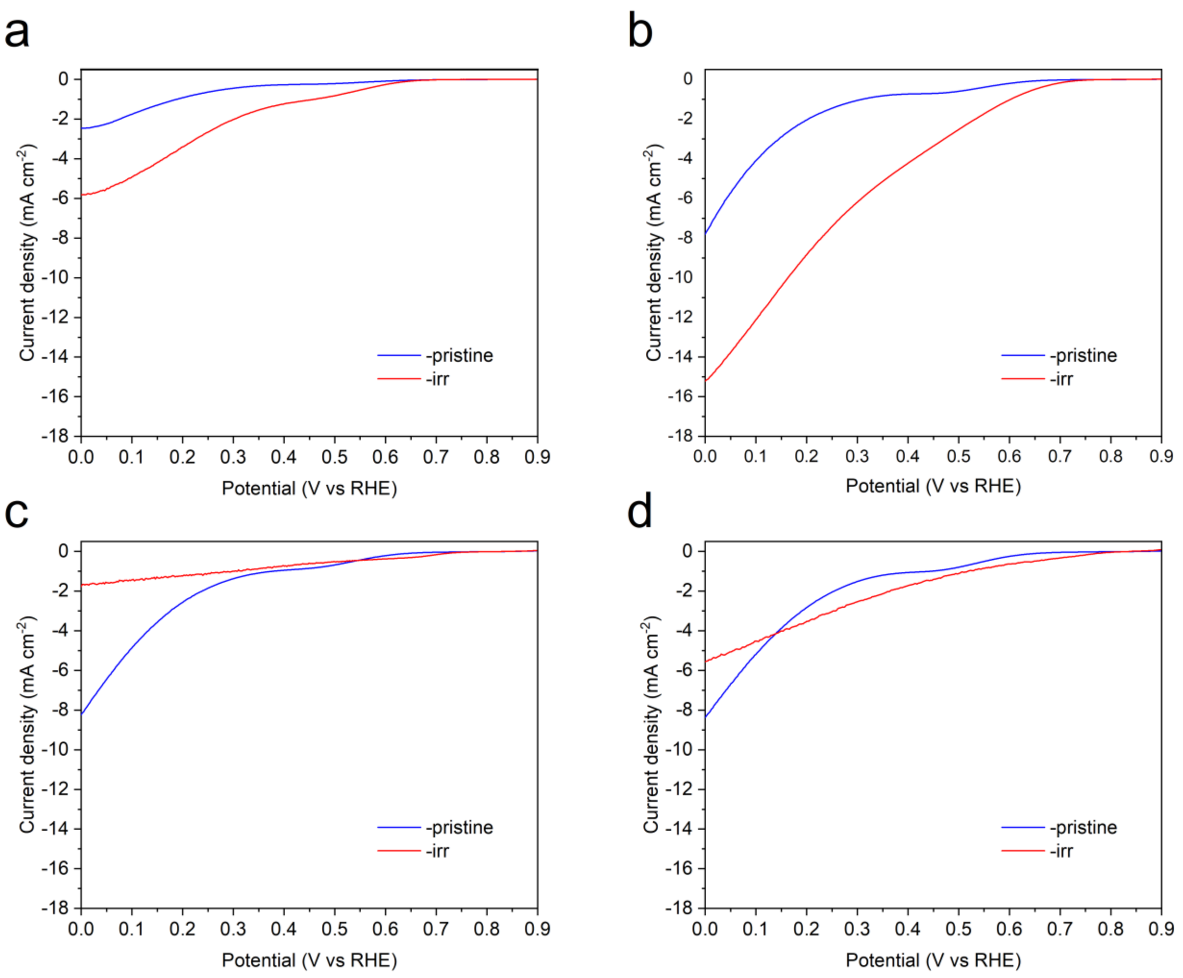

Figure S15. ORR performance for TiN/NF@Toray before (-pristine, blue lines) and after 2 h photo-irradiation (-irr, red lines) for different amounts TiN loading: a) 0.3 mg, b) 1 mg, c) 2 mg, and d) 4 mg. All the measurements were collected in O_2-saturated 0.1 M KOH at a scan rate of 5 mV s^{-1} with a planar film electrode.

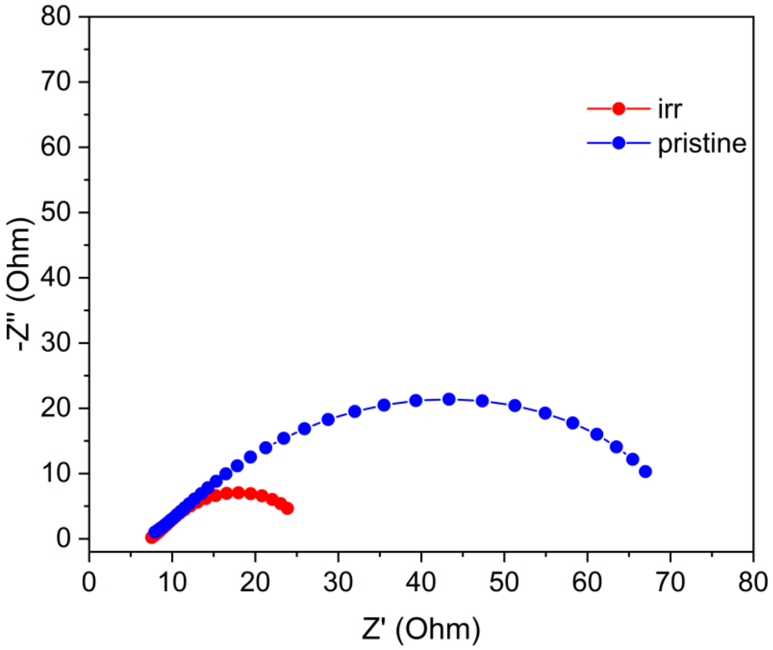

Figure S16. Electrochemical impedance spectroscopy (EIS) of TiN/NF@Toray before (-pristine, blue line) and after irradiation (-irr, red line). The EIS measurement was carried out with amplitude of 10 mv and frequency for 6000 kHz to 1 Hz at 0.3 V vs RHE in 0.1 M KOH after O_2 saturated.

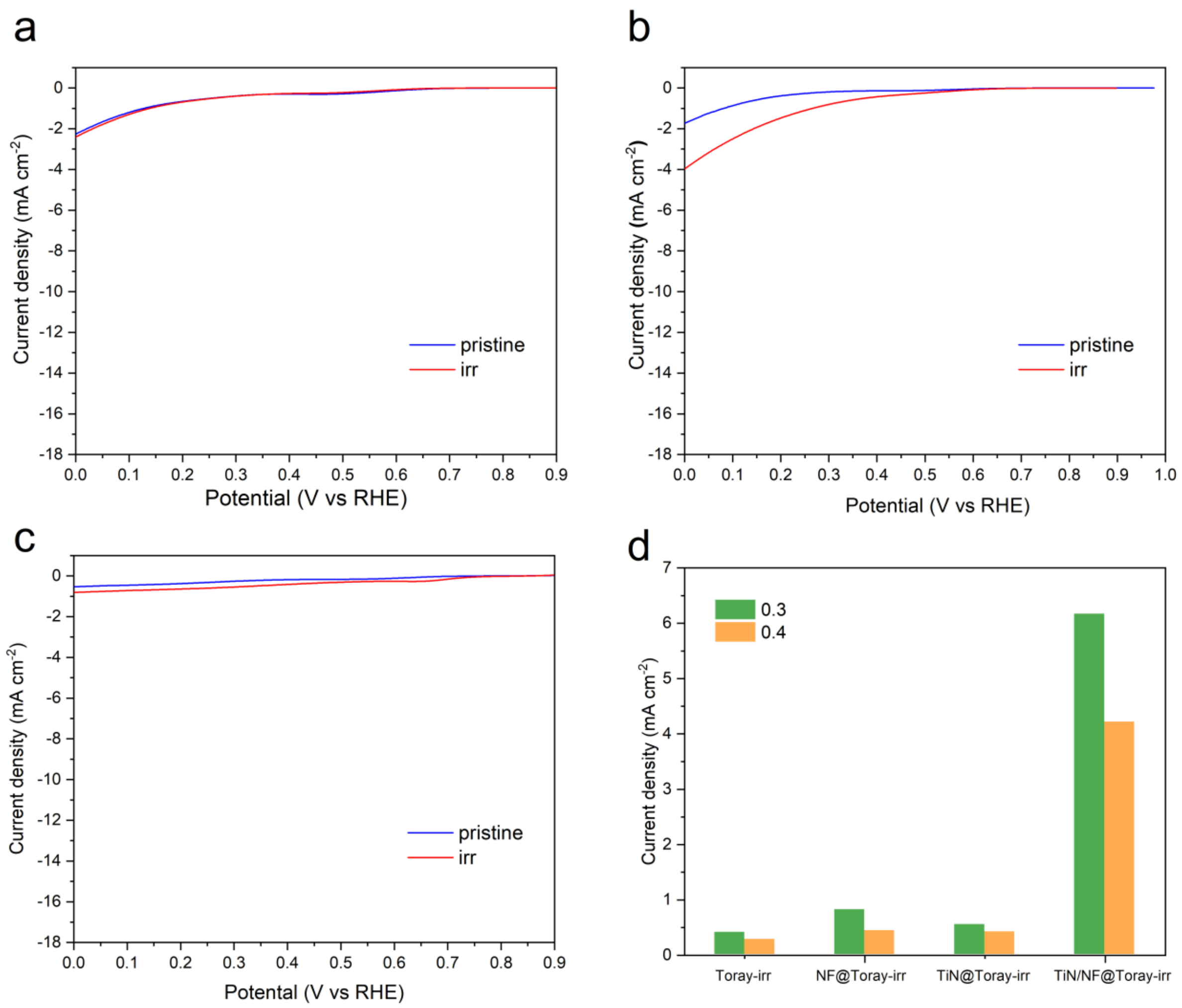

Figure S17. ORR performance in H-cell of a) Toray, b) NF@Toray, and c) TiN@Toray before (-pristine, blue line) and after irradiation (-irr, red line). d) The comparison of current density based on LSV curves at different potentials for the samples after photo-irradiation.

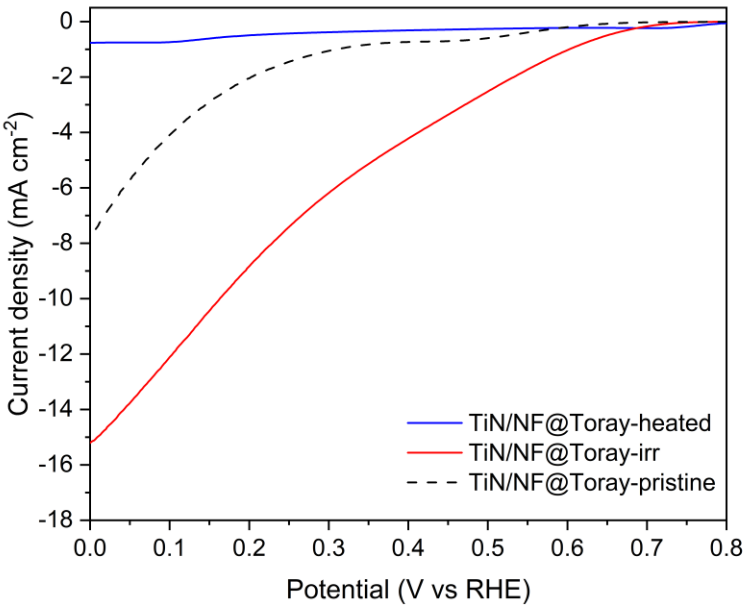

Figure S18. The comparison of ORR performance for TiN/NF@Toray-irr and the sample prepared via pyrolysis in Ar at 430 °C (TiN/NF@Toray_heated), where TiN/NF@Toray-pristine is displayed for reference.

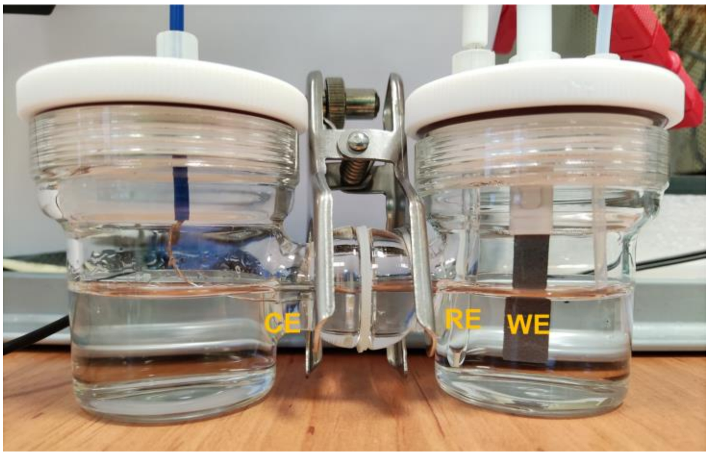

Figure S19. Experimental set-up employed for bulk electrolysis with planar electrodes. The electrochemical H-cell features two different compartments separated by a Nafion 117 membrane. Working electrode (WE) and Ag/AgCl reference electrode (RE) are in one chamber. Pt foil as the counter electrode (CE) is placed in the other chamber.

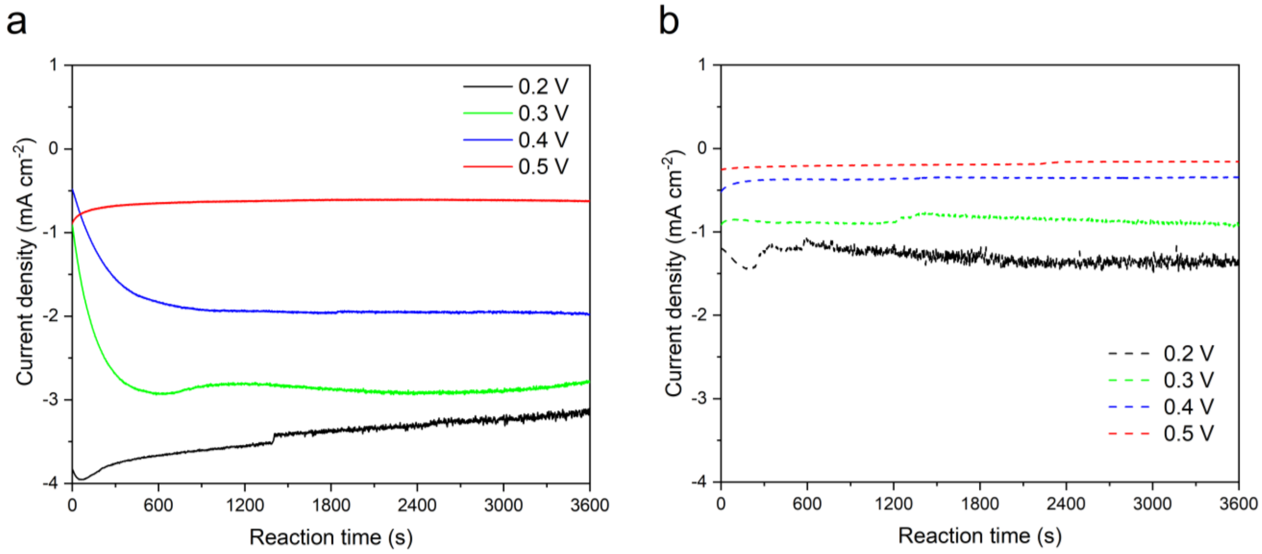

Figure S20. Chronoamperometry measurements in H-cell at different applied potentials: a) the TiN/NF@Toray-irr (solid lines) and b) TiN/NF@Toray-pristine (dashed lines).

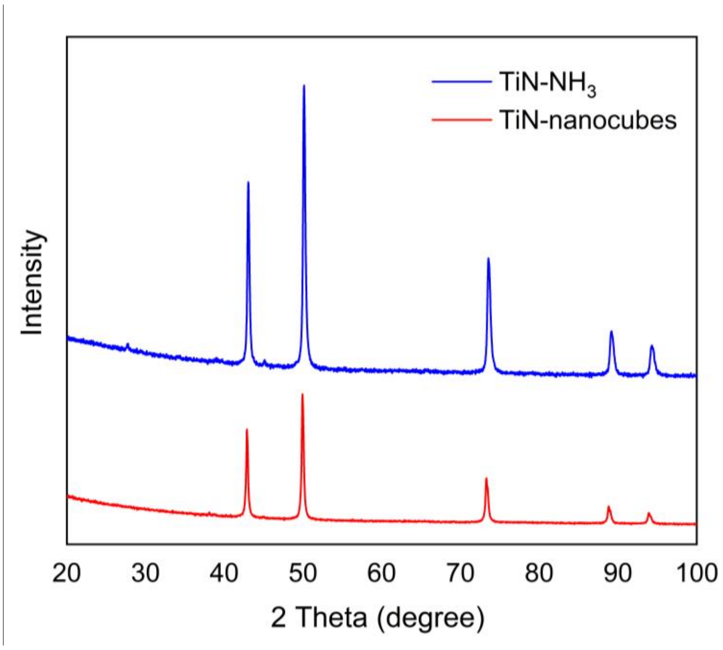

Figure S21. Powder XRD patterns of NH_3 treated commercial TiN nanocubes (TiN-NH_3) samples, where commercial TiN nanocubes (TiN-nanocubes) were used as reference.

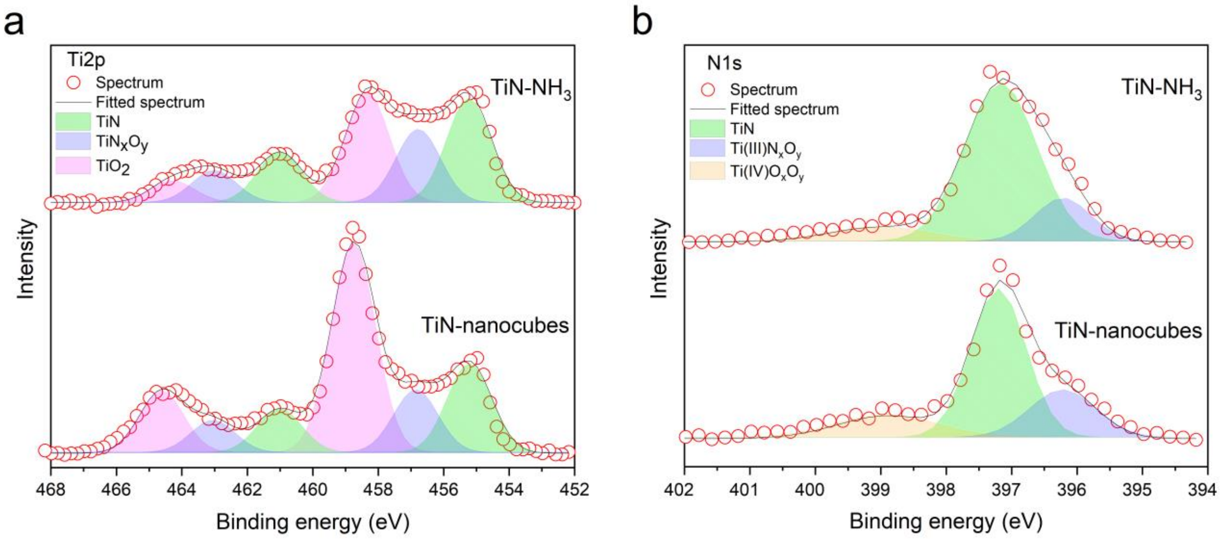

Figure S22. Deconvoluted HR XPS spectra for commercial TiN nanocubes (TiN-nanocubes) and NH_3 treated commercial TiN nanocuebs (TiN-NH_3) in the a) Ti2p and b) N1s regions.

Table S13. The relative amount of the different Ti species according to the deconvolution of HR XPS spectra of Ti 2p spectra for commercial TiN nanocubes (TiN-nanocubes) and NH_3 treated TiN nanocuebs (TiN-NH_3).

C species	*TiN-nanocubes*	*TiN-NH_3*
**TiN (at.%)**	26.5	39.5
**TiO_xN_y (at.%)**	18.9	26.8
**TiO_2 (at.%)**	54.6	33.7

Table S14. The relative amount of the different Ti species according to the deconvolution of HR XPS spectra of N1s spectra for commercial TiN nanocuebs (TiN-nanocubes) and NH_3 treated TiN nanocuebs (TiN-NH_3).

C species	*TiN-nanocubes*	*TiN-NH_3*
**TiN (at.%)**	60.2	74.8
**Ti(III)N_xO_y (at.%)**	16.7	9.5
**Ti(IV)O_xN_y (at.%)**	23.1	15.7

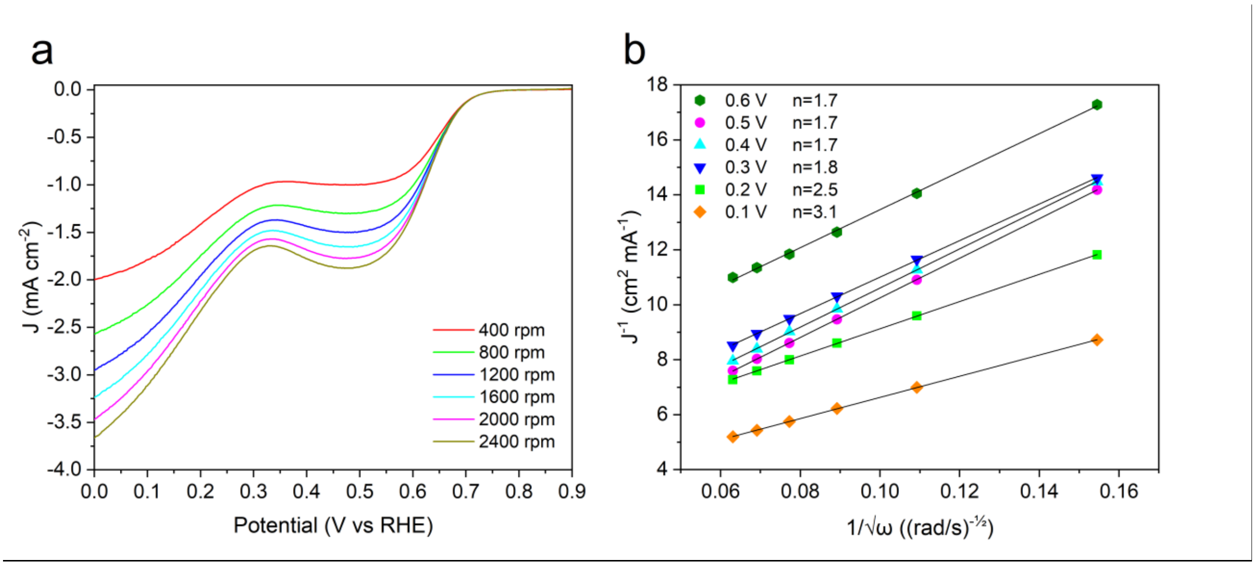

Figure S23. Rotation-rate-dependent voltammograms of commercial TiN nanocubes (TiN-nanocubes) at a scan rate of 5 mV s^{-1} collected using RDE in O_2-saturated 0.1 M KOH. **b)** Koutecky–Levich plots of TiN-nanocubes collected at various potentials (n = calculated number of transferred electrons).

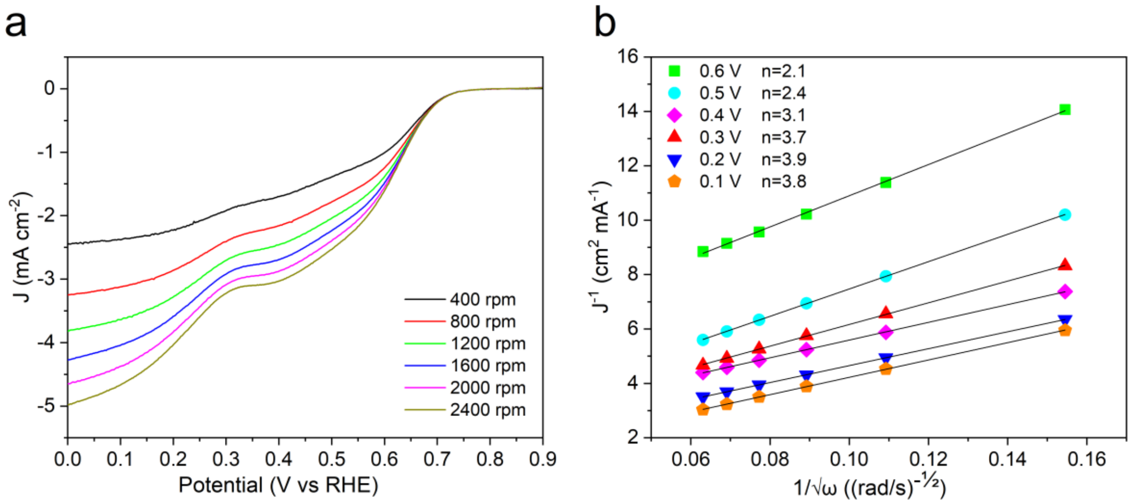

Figure S24. Rotation-rate-dependent voltammograms of NH_3 treated commercial TiN nanocubes (TiN-NH_3) at a scan rate of 5 mV s^{-1} collected using RDE in O_2-saturated 0.1 M KOH. **b)** Koutecky–Levich plots of TiN-NH_3 collected at various potentials (n = calculated number of transferred electrons).

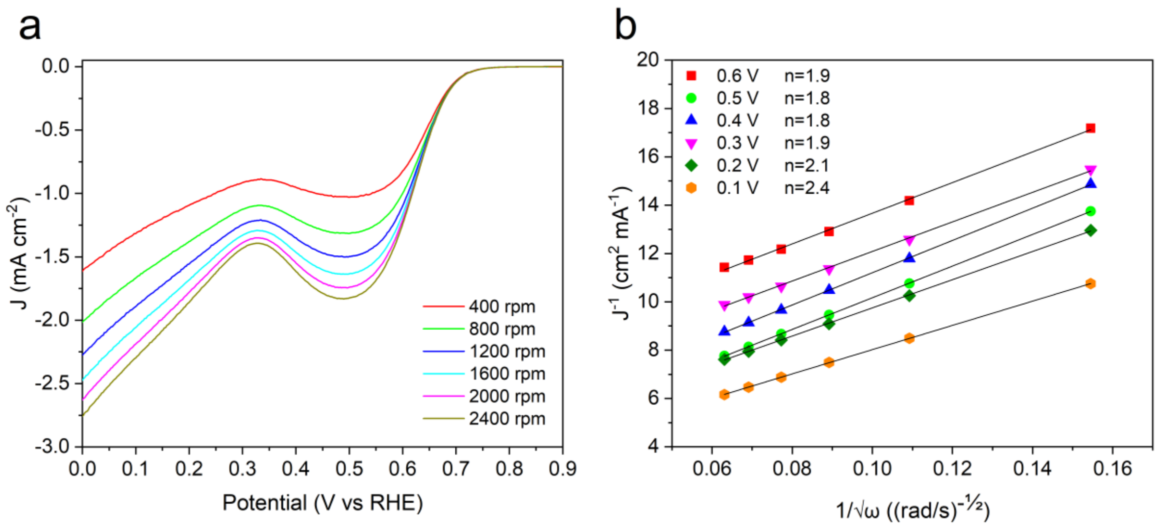

Figure S25. Rotation-rate-dependent voltammograms of TiN particles scraped from the top layer of the TiN/NF@Toray-irr sample at a scan rate of 5 mV s^{-1} collected using RDE in O_2-saturated 0.1 M KOH. **b)** Koutecky–Levich plots of TiN-irr collected at various potentials (n = calculated number of transferred electrons).

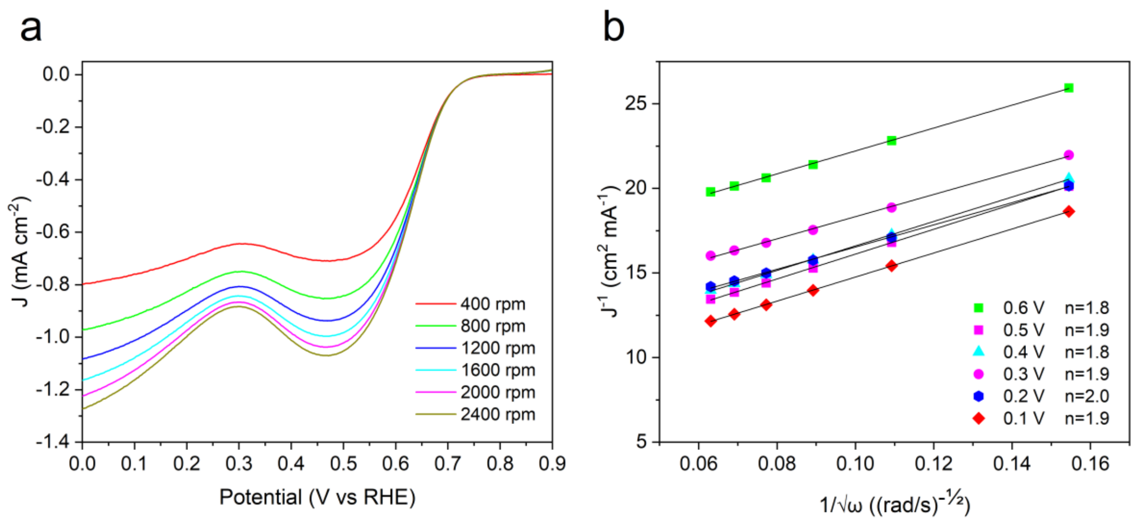

Figure S26. Rotation-rate-dependent voltammograms of commercial TiO_2 nanoparticles (P25 Evonik) performed at a scan rate of 5 mV s^{-1} collected using RDE in O_2-saturated 0.1 M KOH. **b)** Koutecky–Levich plots of TiO_2 collected at various potentials (n = calculated number of transferred electrons).

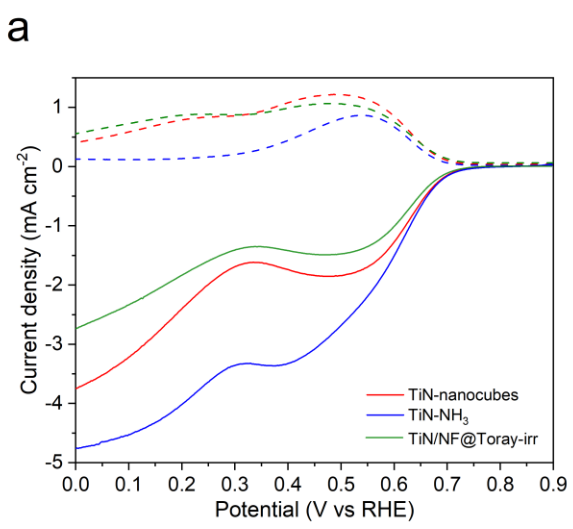

Figure S27. Comparison of ORR activity of commercial TiN nanocubes (TiN-nanocubes), NH_3 treated commercial TiN nanocuebs (TiN-NH_3), and the TiN particles (TiN/NF@Toray-irr) scraped from the top layer of TiN/NF@Toray-irr: a) polarization curves (solid lines) and the current of H_2O_2 production collected on the Pt ring adjusted by collection efficiency (dashed lines), and b) corresponding H_2O_2 selectivity as a function of applied potential. The curves were collected using RRDE in O_2 saturated 0.1 M KOH at a scan rate of 5 mV s^{-1} with a rotation speed of 1600 rpm.

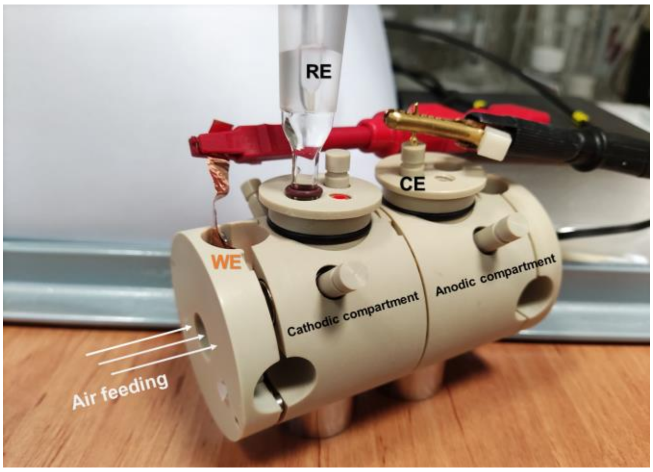

Figure S28. Experimental set-up employed for bulk electrolysis with an air gas diffusion electrode in the cathode compartment. The electrochemical cell is in H-cell configuration with two different compartments separated by Nafion 117 membrane. Particularly, air was let feeding spontaneously from one side of the working electrode (WE) and the other side was in contact with aqueous electrolyte. Ag/AgCl was the reference electrode (RE). Pt wire was used as the counter electrode (CE) is in the other chamber.

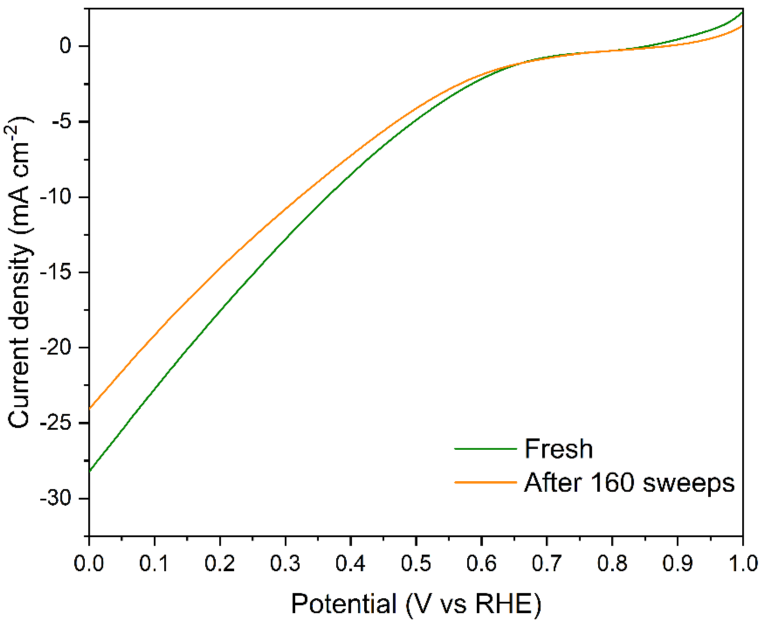

Figure S29. The stability evaluation of TiN/PTFE@Toray-irr the comparison of LSV curves collected with the fresh samples and the one collected after 160 sweeps
